# *Lactiplantibacillus plantarum* inhibited the growth of primary liver cancer by inducing early apoptosis and senescence, *in vitro*

**DOI:** 10.3389/fmicb.2024.1451170

**Published:** 2024-11-11

**Authors:** Oladimeji Paul Duduyemi, Kateryna Potapenko, Nataliia Limanska, Sofiya Kotsyuda, Nataliia Petriv, Huizhen Suo, Tetyana Gudzenko, Volodymyr Ivanytsia, Tetyana Yevsa

**Affiliations:** ^1^Department of Gastroenterology, Hepatology, Infectious Diseases, and Endocrinology, Hannover Medical School, Hanover, Germany; ^2^Department of Microbiology, Virology, and Biotechnology, Odesa I. I. Mechnykov National University, Odesa, Ukraine

**Keywords:** hepatocellular carcinoma, cholangiocarcinoma, lactobacilli, sonicated extracts, probiotics, apoptosis

## Abstract

Primary liver cancer (PLC), comprising hepatocellular carcinoma (HCC) and cholangiocarcinoma (CCA), is a severe form of cancer associated with a high mortality and morbidity rate and increasing incidence worldwide. Current treatment options are limited and chemotherapeutics demonstrate strong side effects. New therapies are highly required. Lactobacilli represent the most diverse lactic acid-producing bacteria group and a prominent example of probiotics. Several studies have highlighted the anticancer efficacy of probiotics, especially of *Lactiplantibacillus plantarum*. However, there are limited studies on its activity on two PLC types, hepatocellular carcinoma (HCC) and cholangiocarcinoma (CCA). This study evaluated the inhibitory mechanism and properties of *L. plantarum* ONU 12 (Lp 12) and *L. plantarum* ONU 355 (Lp 355), isolated from grapes in Ukraine and France, in murine PLC cell lines, *in vitro.* Strain *Lacticaseibacillus casei* ATCC 393 (Lc 393) has been taken for a direct comparison, as the most studied probiotic strain. The three *Lactobacillus* species were used in three forms: as live and heat-killed suspensions, and as sonicated extracts, and tested either as a monotherapy or in combination with standard chemotherapeutics (sorafenib for HCC and gemcitabine for CCA). Cell proliferation and viability were assessed via crystal violet staining assay and cell counting kit-8 assay. The induction of senescence was investigated by senescence-associated β-galactosidase assay. Fluorescence-activated cell sorting analysis was used to determine the apoptotic mechanism behind the inhibitory property of lactobacilli. The results showed that the live suspensions and sonicated extracts of Lp 12, Lp 355, and Lc 393 demonstrated inhibitory properties in CCA and HCC cells after 48 h of incubation. In combinations with standard chemotherapeutics, lactobacilli treatments have shown strong synergistic effects. The combination therapy allowed to reduce the chemotherapeutic doses of gemcitabine from 50 μM to 0.1 and 0.05 μM and sorafenib from 13.8 μM to 6.9 and 3.45 μM. Successful treatment regimes induced early apoptosis and cellular senescence in PLC, as the mechanism of inhibition. Heat-killed suspensions showed no inhibitory effect in none of the cell lines. Both strains, Lp 12 and Lp 355, showed successful results and need further testing *in vivo*, using autochthonous HCC and CCA models.

## 1 Introduction

Primary liver cancer (PLC) is a severe form of cancer, associated with a high mortality and morbidity rate worldwide. In 2020, it accounted for an estimated 830,180 deaths and 905,677 new cases globally, making it the third leading cause of cancer-related death, its incidence has been rising in many countries and is projected to continue increasing in future (Liu et al., 2022). Hepatocellular carcinoma (HCC) and cholangiocarcinoma (CCA) are the most common leading types of PLC, accounting for 70–85% HCC and 10–20% CCA of all liver cancer cases, respectively (Banales et al., 2020; El-Serag and Rudolph, 2007). HCC stems from the liver cells (mature hepatocytes), while CCA arises from the cholangiocytes of the biliary ducts (Gromowski et al., 2023). The main factors that increase the risk of liver cancer are persistent viral infection of hepatitis B and C, alcoholic liver diseases, exposure to aflatoxins, metabolic dysfunction-associated steatotic liver disease, and metabolic disorders such as obesity and diabetes (Chan et al., 2023; Forner et al., 2018; Rawla et al., 2018). These conditions lead to liver inflammation, fibrosis, and cirrhosis and eventually initiate the progression to cancerous cells. The prevalence of liver cancer varies by geographical region based on the risk mentioned above, with the highest rate observed in Asia and sub-Saharan Africa (Sung et al., 2021). The available treatment options include surgical resection, liver transplantation, chemotherapy, radiation therapy, targeted therapy, and immunotherapy (Jiang et al., 2023; Suresh et al., 2023). However, the treatment choice for liver cancer depends on diverse factors such as the size, stage, and location of the tumor and the patient’s overall health; for example, surgery is the most preferred treatment option for early-stage patients where the tumor is localized and not gone to metastasis and can be removed (Liu et al., 2015; Naqi et al., 2014). However, despite the availability of numerous treatment options, the clinical outcome does not directly correspond to the effort invested in eliminating liver cancer. These treatment options especially chemotherapy has severe side effects and a high recurrence rate (Llovet et al., 2005). As a result, it is imperative to intensify research to develop new alternative and complementary treatment strategies to improve prognosis, reduce the severe side effects of existing therapies and reduce the global burden of PLC.

A promising approach is the correction of the gut microbiome with beneficial microorganisms or treatment with their metabolites (Temraz et al., 2021). It is known that probiotics confer health benefits when administered in the correct concentration (Swanson et al., 2020). Research has examined the various roles of probiotics and their potential to prevent and treat various conditions, including diarrhea, cancer, hypertension, autism, and migraines (Liu et al., 2018; Tegegne and Kebede, 2022). These beneficial effects have garnered significant attention. An important example of such probiotics is *Lactiplantibacillus plantarum*, which has been shown to possess many properties such as immune modulation, anti-oxidative, anti-inflammatory, regulation of glucose levels, and metabolic pathways (Liu et al., 2018). *L. plantarum* belongs to the genus *Lactobacillus,* the most diverse lactic acid-producing bacteria (LAB) group (Seddik et al., 2017). *L. plantarum* is a Gram-positive, short rod, non-spore-forming, non-pathogenic, and probiotic bacterium naturally found in the human microbiome (Kazmierczak-Siedlecka et al., 2020). It is also found in dairy products (Agaliya and Jeevaratnam, 2012), meat (Schillinger and Lucke, 1989), and wine (Berbegal et al., 2016). The ubiquitous nature of *L. plantarum* proves its ability to survive and adapt to extreme environmental conditions, even in the acidic environment of the stomach and bile salt in the small intestine (Fiocco et al., 2010). LAB inhibits the growth of pathogens like *Staphylococcus aureus, Listeria monocytogenes, Salmonella enteriditis*, and *Escherichia coli* (Oldak et al., 2020; Oldak et al., 2017). They exert these antibacterial effects by producing bacteriocins, organic acids and by directly competing for nutrients and adhesion sites to prevent colonization and growth of pathogenic bacteria (Mokoena, 2017). Different species of lactobacilli, including *L. acidophilus*, *L. casei*, *L. rhamnosus*, and *L. plantarum* have proven to be associated with the lowering of serum cholesterol and fibrinogen and preventing cardiovascular diseases in humans (Anderson and Gilliland, 1999).

Based on above mentioned, in our study we focused on *L. plantarum.* We have chosen two strains of *L. plantarum* (ONU 12 and ONU 355) isolated from the must of grape in Ukraine and France, which have been demonstrated to have strong bacteria antagonistic activity and ability to form biofilms (Limanska et al., 2019). We tested the selected *L. plantarum* strains for their anti-cancerous potential against HCC and CCA as complementary or alternative therapy to the standard chemotherapeutics. We directly compared the efficacy of two strains *L. plantarum* to the most studied lactobacilli strain, *Lacticaseibacillus casei* ATCC 393 known for its anticancer effect (Lau et al., 2024; Spyridopoulou et al., 2021).

## 2 Materials and methods

### 2.1 Cultivation of bacterial strains

*L. plantarum* strains ONU 12 and ONU 355 were initially isolated from must of grape gathered in Ukraine and France (Limanska et al., 2019). *L. casei* ATCC 393 known for its anti-cancer effect described in the literature (Lau et al., 2024; Spyridopoulou et al., 2021) and purchased from the DSMZ, Leibniz Institute, Braunschweig, Germany (DSMZ No.: 20011) was used as a control. *L. plantarum* and *L. casei* were cultivated in de Man Rogosa and Sharpe (MRS) medium (Haghshenas et al., 2023) at 37°C aerobically_._

### 2.2 Preparation of live suspensions of bacteria

Lactobacilli strains were cultivated in MRS broth at 37°C for 20–24 h to reach a concentration of at least 10^9^ CFU/mL. Bacterial cells were washed twice in PBS buffer and harvested by centrifugation at 3,500 g for 15 min at 4°C (S. Choi et al., 2006). Dulbecco’s Modified Eagle’s Medium (DMEM, Gibco, UK) without antibiotics was used to adjust the obtained bacterial pellet to a starting concentration of 10^9^ CFU/mL.

### 2.3 Preparation of heat-killed suspensions of bacteria

Lactobacilli were grown in MRS broth at 37°C and allowed to reach a concentration of at least 10^9^ CFU/mL. Thereafter, bacterial suspensions were harvested and washed twice in PBS as described in the previous section. The bacterial pellet was resuspended in PBS and distributed into 2 mL microtubes. Using a thermoblock (Eppendorf, USA), heat inactivation was performed at 95°C for 1 h (Choi et al., 2006) and vortexed intermittently to ensure a complete inactivation. Thereafter, lactobacilli were centrifuged again at 3,500 *g* for 15 min at 4°C and resuspended in DMEM without antibiotics.

### 2.4 Preparation of sonicated extracts of bacteria

Sonication was performed to break apart or disrupt the bacterial cell wall and membrane to release bacterial protein using sound energy (Wang et al., 2021). Lactobacilli were cultivated in MRS broth at 37°C for 20–24 h to reach a concentration of at least 10^9^ CFU/mL. Bacterial suspensions were harvested and washed twice in PBS buffer using the same conditions as described in the previous sections. Thereafter, 1 mL of bacterial suspensions were distributed into several 1.5 mL microtubes and disrupted in a sonic dismembrator according to Han et al. (2013) with modifications: 10 rounds for 1 min each at 70 W. Afterward, the sonicated extracts were collected into a falcon and two-fold dilution with DMEM without antibiotics was prepared for experiments and subjected to protein quantification by Pierce™ BCA protein assay kit (Thermo Scientific™, USA), according to the manufacturer’s instructions.

### 2.5 Cell lines and their maintenance

Cancer cell lines, HCC expressing *NRAS^G12V^*oncogene and CCA expressing *KRAS^G12V^*and *Akt2* oncogenes, isolated from murine PLC as described recently (Pylypchuk et al., 2022; Suo et al., 2022) were used in this study. Commercial murine fibroblasts CBA-310 (Zhang et al., 2023) were used as control non-cancerous cells. All cell lines were cultivated in a complete DMEM supplemented with 10% fetal bovine serum, 5% penicillin/streptomycin, and 5% minimum essential medium non-essential amino acids (Thermo Fisher, USA). The cells were grown in the incubator at 37°C, with 5% CO_2_ and 95% humidity.

### 2.6 Treatment of HCC, CCA cells and fibroblasts with lactobacilli

Cells were first seeded at concentration 1 × 10^5^/mL in 100 μL volume in complete DMEM medium without antibiotics. After 16 h of incubation, 100 μL of twice concentrated live suspension or sonicated extracts or heat-killed suspensions were added into each well of the 96-well plates accordingly. Also, standard anticancer therapeutics sorafenib (in case of HCC) and gemcitabine (in case of CCA) were administered as monotherapy or in combination with *L. plantarum* suspensions or extracts. Half (6.9 μM) dose and quarter (3.5 μM) doses of the clinical plasma concentration of sorafenib (13.8 μM) were used, as described recently (Pylypchuk et al., 2022; Suo et al., 2022). For gemcitabine, a small fraction of its plasma concentrations, specifically 0.05 μM and 0.1 μM, derived from the clinical plasma concentration of gemcitabine (50 μM) were used, as described recently (Pylypchuk et al., 2022; Suo et al., 2022). Sonicated PBS, PBS, and DMEM were used as negative controls. Subsequently, the plates were incubated for 24 h and 48 h for further experimentation and analysis.

### 2.7 Bright field microscopy

Cell monolayers were observed and photographed using a Nikon Eclipse Ti2 microscope with a 40x objective.

### 2.8 Crystal violet staining assay (CVSA)

The evaluation of cancer cell growth was conducted using CVSA assay employing a well-established standard technique as described in the works of Śliwka et al. (2016), Pylypchuk et al. (2022), and Suo et al. (2022). Briefly, following a 24 and 48 h incubation period, the cells were gently washed with warm 100 μL PBS to remove residual DMEM or debris. Subsequently, the cells were fixed using a 4% paraformaldehyde solution for 7 min, ensuring the cells were adequately immobilized. Once fixed, the cells were stained with 0.5% crystal violet in 30% ethanol for 30 min at room temperature. The staining dye binds to the cellular component of live cells allowing for optimal visualization and quantification of cell density. The plates were then left to dry overnight. Finally, an ImmunoSpot® S6 ULTI-MATE Analyzer (Cellular Technology Limited, USA) was employed to capture high-resolution photographs of each plate well and analyzed using ImageJ software.

### 2.9 Cell proliferation assay/cell counting kit-8 (CCK-8)

The CCK-8 method was employed to quantitatively assess cell viability and proliferation in a precise and objective manner, as established (Pylypchuk et al., 2022; Suo et al., 2022). The experimental procedure was carried out according to the manufacturer’s protocol to ensure standardization and accuracy. After 24 and 48 h incubation periods, 10 μL of the CCK-8 solution was carefully added to each well of the plate and further incubated for 2–4 h. During this time, the water-soluble tetrazolium salt present in the CCK-8 reagent underwent an enzymatic reduction in viable cells, generating a colored formazan product (Yang et al., 2021). To quantify this cellular response, the absorbance of each well was measured after 2 and 4 h at a wavelength of 450 nm, utilizing the Infinite 200 PRO Nano Quant Tecan Microplate Reader (TECAN, Switzerland). In CCK-8 assay, the intensity of the formazan product and the optical density readings directly correlates with the abundance of viable cells present in the well (Yang et al., 2021). The resulting absorbance values were subsequently analyzed using the i-control™ software (TECAN, Switzerland).

### 2.10 Senescence-associated beta-galactosidase assay (SA-β-gal)

Cellular senescence was evaluated by SA-β-Gal assay, as established (Pylypchuk et al., 2022; Suo et al., 2022), following a 24 and 48 h incubation period of cells with the tested lactobacilli and their extracts. Initially, the cell monolayers were gently washed with PBS at a pH range of 7.2–7.4 to eliminate any residual media or debris. After, the cells were fixed with a fixation solution comprising 2% formaldehyde and 0.2% glutaraldehyde in PBS at the aforementioned pH range. Following the removal of the fixation solution, the cells underwent an additional wash step with PBS buffer at pH 6.0 supplemented with 1 mM MgCl_2_. Hundred μL of X-gal staining solution was added to each well. The staining solution, prepared according to the protocol described by Cahu and Sola, (2013), consisted of potassium ferrocyanide, potassium ferricyanide, and X-galactose in PBS supplemented with 1 mM MgCl_2_ at pH 6.0 (Cahu and Sola, 2013). The plates were then incubated at 37°C without CO_2_ supply until a distinct blue stain developed, indicating the presence of senescent cells. To capture representative images of analysis, ten high-power field photos were taken from each well using a Nikon microscope Eclipse Ti2 (Nikon, Japan) at objective 40x. The images were subsequently analyzed to assess the extent of cellular senescence induced by the lactobacilli and their extracts.

### 2.11 Flow cytometry analysis (FACS) to detect early, late apoptosis and necroptosis

The cells, following a 24 and 48 h incubation were carefully harvested and washed with PBS at pH 7.2–7.4 ranges. To detach the adherent cells, trypsin was added and the resulting cell suspension was transferred into the initial microtubes, respectively. For the assessment of apoptosis, a dual staining approach was implemented using Annexin V-Phycoerythrin (PE) and 7-Amino-Aactinomycin D (7-AAD) (Biolegend^®^, USA), as described (Bushnell, 2015). Annexin V-PE binds specifically to phosphatidylserine, which translocates from the inner to the outer leaflet of the plasma membrane during early apoptosis (Vermes et al., 1995). 7-AAD, a DNA-binding dye, is impermeable to intact cell membranes but stains the DNA of late apoptotic and necrotic cells with compromised membrane integrity (Zembruski et al., 2012). Five μL of Annexin V-PE and 7-AAD stains were added, respectively, to the cell suspension. A 400 μL of binding buffer consisting of 10 mM HEPES [pH 7.4], 140 mM NaCl, and 2.5 mM CaCl_2_ was added to each tube. The samples were then subjected to FACS analysis using a flow cytometer (Cytek® Aurora, USA). Compensation was performed and the acquired data were subsequently analyzed using FlowJo 9.9.6 (BD™, USA) software.

### 2.12 Statistical analysis

All graphs were plotted using GraphPad Prism (GraphPad Prism 10, USA) software. All CVSA plate pictures were carefully analyzed by ImageJ software and extracted data were further analyzed by GraphPad Prism to plot respective graphs. The experiment was carried out in triplicate and two-way ANOVA was used for all statistical analyses to calculate significant differences among experimental and control groups. If not stated otherwise, data are shown as mean ± standard error of the mean (S.E.M.) with *p* < 0.05 considered statistically significant. Significance levels were depicted as *p < 0.05, **p < 0.01, ***p < 0.001, and ****p < 0.0001.

## 3 Results

**Figure 1:**
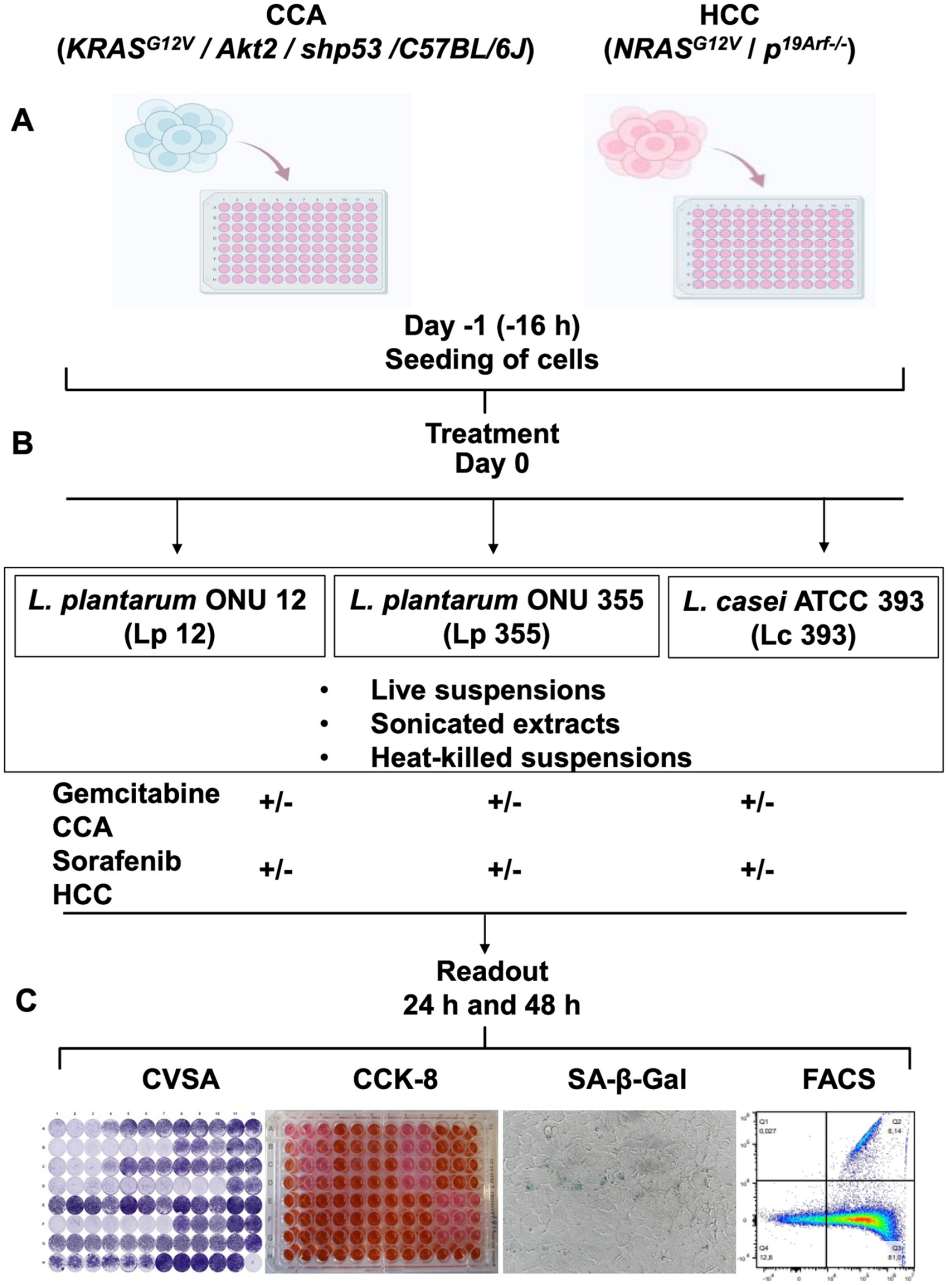
Experimental layout to study the efficacy of Lp 12, Lp 355, and Lc 393, and the mechanism of their action *in vitro* using CCA, HCC cell lines and fibroblasts. **(A)** Cells were seeded on day −1; **(B)** on day 0, live, heat-killed suspensions, and sonicated extracts of either Lp 12 or Lp 355 or Lc 393 were added to the cells at different concentrations as monotherapy or in combination with standard chemotherapeutics, gemcitabine for CCA and sorafenib for HCC. **(C)** After 24 and 48 h of incubation, different readouts were performed, comprising CVSA, CCK-8, SA-β-Gal assays, and FACS analysis, as well as bright field microscopy. Commercial murine fibroblasts CBA-310 were used as a control cell line (normal cells).

Three different lactobacilli strains were used in this study: *L. plantarum* ONU 12 (Lp 12) and *L. plantarum* ONU 355 (Lp 355) which were isolated from the must of grape (Limanska et al., 2019), and lastly, *L. casei* ATCC 393 (Lc 393) which was purchased from the DSMZ, Germany. All bacteria were grown in MRS broth for at 37°C for 20–24 h and prepared as live suspensions, or sonicated extracts, or heat-killed suspensions, for use in this study. The HCC and CCA cell lines were established previously (Pylypchuk et al., 2022; Suo et al., 2022) and seeded in a 96-well plate (Figure 1A). After 16 h, the live suspensions, sonicated extracts, and heat-killed suspensions of Lp 12, Lp 355, and Lc 393 in combinations with the respective standard chemotherapeutic drugs (sorafenib for HCC and gemcitabine for CCA) were added and co-incubated with the already seeded cells (Figure 1B). Importantly, the standard therapeutics were added at different reduced concentrations. Positive controls comprised a clinical plasma concentration of sorafenib (13.8 μM) and a clinical plasma concentration of gemcitabine (50 μM), as established previously (Pylypchuk et al., 2022; Suo et al., 2022). Negative controls comprised the diluent/buffers and DMEM. 24 and 48 h post-incubation, assays such as CVSA, CCK-8, SA-β-Gal, FACS analysis, and bright field microscopy were carried out to assess the inhibitory properties and mechanism of action of the treatment added (Figure 1C).

### 3.1 Anticancer activity screening in CCA cells

#### 3.1.1 Live suspension and sonicated extracts of Lp 12 and Lp 355 as monotherapy or in combination with gemcitabine inhibited the growth of CCA

**Figure 2:**
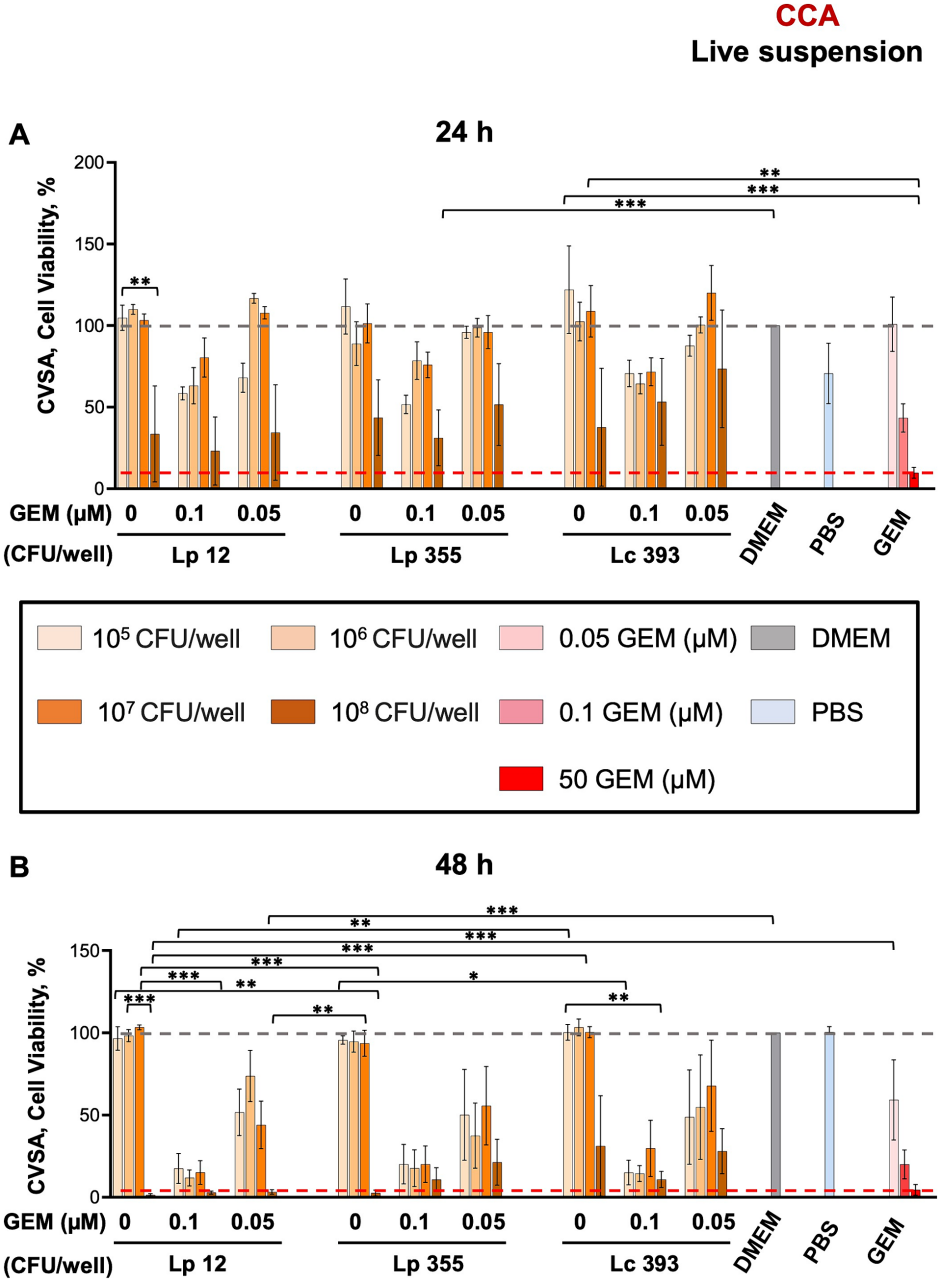
The live suspensions of Lp 12 and Lp 355 at the concentration of 10^8^ CFU/well inhibited the growth of CCA. CVSA analysis was performed on CCA cells after the treatment with lactobacilli administered either alone at different concentrations (live suspension: 10^8^, 10^7^, 10^6^, 10^5^ CFU/well) or in combination with gemcitabine at different concentrations (0.05 and 0.1 μM). CVSA readouts were performed: **(A)** live suspension after 24 h and **(B)** live suspension after 48 h post-incubation. GEM, gemcitabine. The red dashed line shows the data obtained in the control group treated with the standard therapy (gemcitabine 50 μM). *p < 0.05, **p < 0.01, ***p < 0.001.

The inhibitory capacity of live suspensions of Lp 12, Lp 355, and Lc 393 were first evaluated using CVSA assay in CCA cell line. The highest concentration of live suspension (10^8^ CFU/well) of all strains used demonstrated a mild inhibitory effect on CCA after 24 h (Figure 2A), that became more evident after 48 h (Figure 2B) when compared to untreated control (DMEM). Gemcitabine was used as a positive control (50 μM) and as expected, inhibited CCA cells in a dose-dependent manner. The inhibitory effect of the live suspensions of Lp 12, Lp 355, and Lc 393 was also found to be concentration-dependent, with the highest concentration (10^8^ CFU/well) displaying the strongest effect, comparable to the human plasma concentrations of gemcitabine (50 μM). Monotherapy with the highest concentration (10^8^ CFU/well) of Lp 12 and Lp 355 showed stronger inhibitory efficacy than the one in Lc 393 group (Figure 2B). Control strain Lc 393 (10^8^ CFU/well) has demonstrated a toxicity effect on fibroblasts both at 24 h (Supplementary Figures S1A,B) and especially at 48 h (Supplementary Figures S1C,D). Importantly, the highest concentration (10^8^ CFU/well) of Lp 12 and Lp 355 inhibited the growth of CCA, but not of fibroblasts (Figure 2 and Supplementary Figure S1). The inhibitory effect observed in the combination therapies in CCA and fibroblasts was similar to the effects seen in the control groups treated with gemcitabine (0.1 and 0.05 μM) (Figure 2 and Supplementary Figure S1).

**Figure 3:**
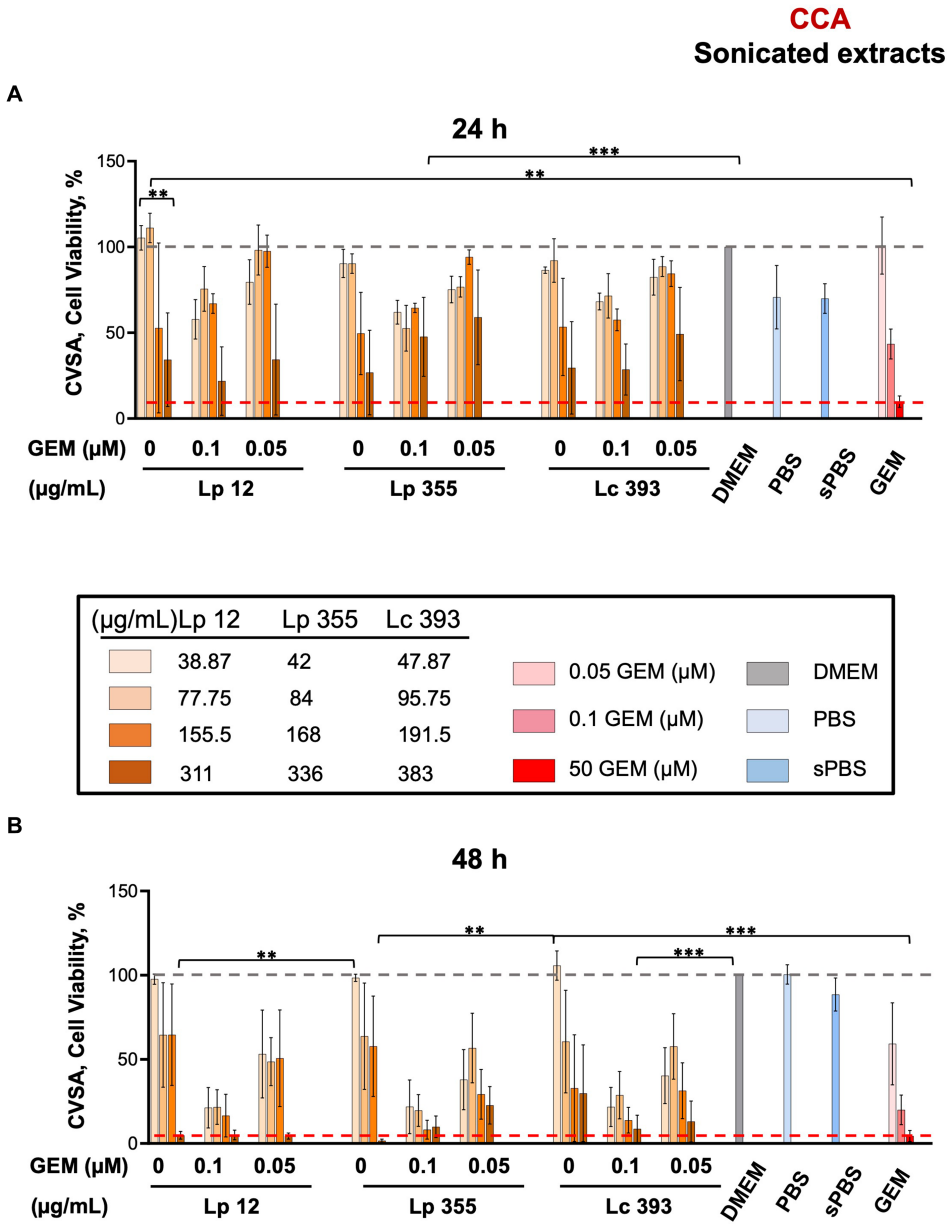
The sonicated extracts of Lp 12 and Lp 355 at the concentrations of 311 and 336 μg/mL, respectively, inhibited the growth of CCA. CVSA analysis was performed on CCA cells after the treatment with lactobacilli administered either alone at different concentrations (sonicated extracts: in the range of 35–390 μg/mL) or in combination with gemcitabine at different concentrations (0.05 and 0.1 μM). CVSA readouts were performed: **(A)** sonicated extracts after 24 h and **(B)** sonicated extracts after 48 h post-incubation. GEM, gemcitabine; sPBS, sonicated PBS. The red dashed line shows the data obtained in the control group treated with the standard therapy (gemcitabine 50 μM). **p < 0.01, ***p < 0.001.

We further tested the sonicated extracts obtained from live bacteria in CCA settings. Similar to live suspensions, the sonicated extracts of Lp 12 (311 μg/mL) and Lp 355 (336 μg/mL) inhibited the growth of CCA cells specifically after 48 h of incubation (Figures 3A,B), as compared to the positive control (gemcitabine 50 μM). Lc 393 (383 μg/mL) showed less inhibitory efficacy, than the two Lp counterparts (Figure 3B). The inhibitory effect observed in the combination therapies was more pronounced than the effects seen in the control groups treated with gemcitabine (0.1 μM and 0.05 μM), especially after 48 h of incubation (Figure 3B). In particular, Lp 12 (311 and 155.5 μg/mL), Lp 355 (336 and 168 μg/mL), and Lc 393 (383 and 191.5 μg/mL) in combination with gemcitabine 0.1 μM performed more efficacious than gemcitabine 0.1 μM alone or sonicated extracts administered as monotherapy (Figure 3B). Furthermore, all tested sonicated extracts in combination with gemcitabine 0.5 μM performed more efficacious than gemcitabine 0.5 μM alone or sonicated extracts administered as monotherapy (Figure 3B). Negative controls – sonicated PBS, PBS, and DMEM – were not able to inhibit the growth of CCA (Figure 3).

When compared to the normal cells fibroblasts, the sonicated extracts of Lc 393 (383 and 191.5 μg/mL) proved to be toxic, whereas Lp 12 and Lp 355 did not demonstrate high toxicity toward fibroblasts (Supplementary Figures S2A–D).

#### 3.1.2 Heat-killed suspensions of Lp 12, Lp 355, and Lc 393 showed no inhibitory effect on CCA

**Figure 4:**
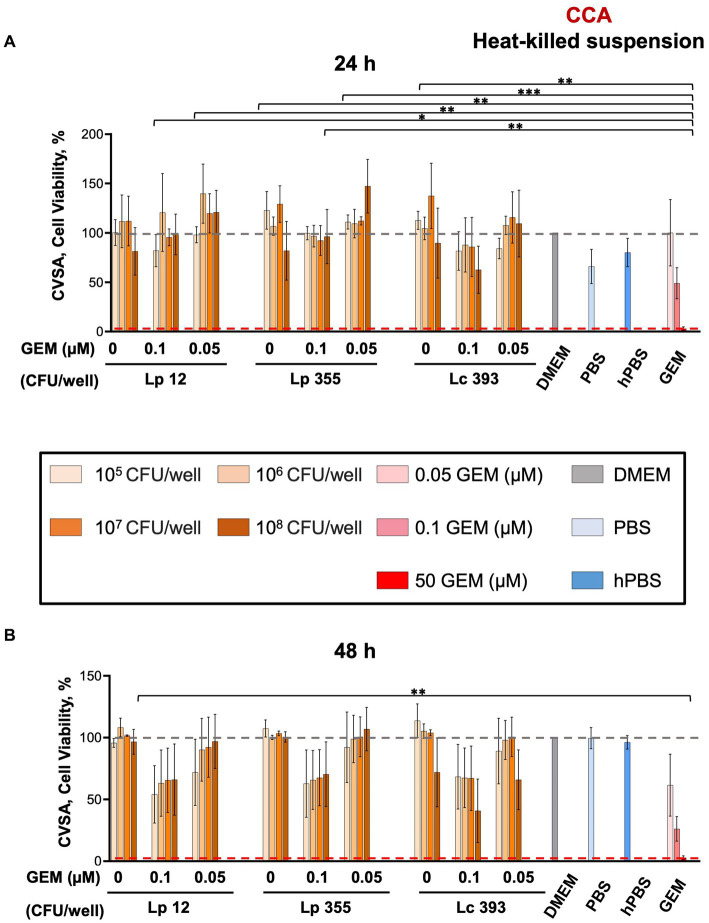
The heat-killed suspensions of Lp 12, Lp 355, and Lc 393 did not inhibit the growth of CCA cells. CVSA analysis was performed on CCA cells after the treatment with lactobacilli administered either alone at different concentrations or in combination with gemcitabine at different concentrations (0.05 and 0.1 μM). CVSA readouts were performed **(A)** 24 h and **(B)** 48 h post-incubation. GEM, gemcitabine; hPBS, heated PBS. The red dashed line shows the data obtained in the control group treated with the standard therapy (gemcitabine 50 μM). *p < 0.05, **p < 0.01, ***p < 0.001.

We further assessed the efficacy of heat-killed suspensions of Lp 12, Lp 355, and Lc 393 in CCA using CVSA assay. Interestingly, we did not observe any impact of heat-killed suspensions and even the highest concentration of 10^8^ CFU/mL of lactobacilli could not inhibit the growth of CCA cells at both 24 and 48 h (Figures 4A,B).

#### 3.1.3 Combination of either sonicated extracts or live suspension of Lp 12, Lp 355, and Lc 393 with gemcitabine inhibited CCA growth

**Figure 5:**
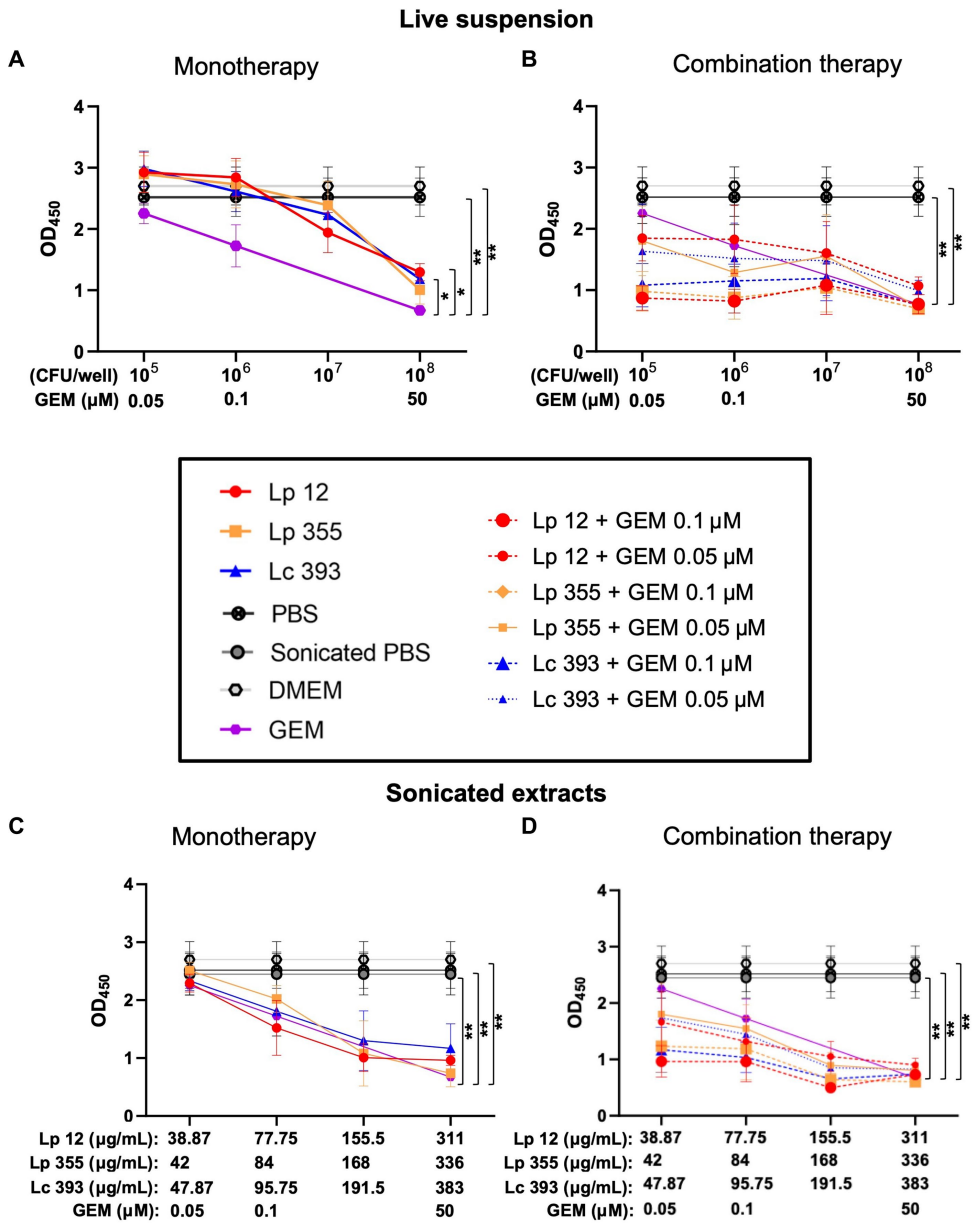
Live suspensions or sonicated extracts of Lp 12, Lp 355, and Lc 393 and their combinations with gemcitabine demonstrated inhibitory effects on CCA cell line in CCK-8 assay. CCA cells were treated with lactobacilli and their combination with gemcitabine: **(A)** 48 h live lactobacilli suspension monotherapy; **(B)** 48 h live lactobacilli suspension monotherapy and combination therapy; **(C)** 48 h sonicated extract lactobacilli monotherapy; **(D)** 48 h sonicated lactobacilli extract monotherapy and combination therapy. After the incubation, CCA cells were subjected to CCK-8 analysis at OD_450_. GEM, gemcitabine. *p < 0.05, **p < 0.01.

In our next step, we performed a CCK-8 assay to quantify the viable cells remaining after the treatment of CCA cells with the live suspension and sonicated extracts of Lp 12, Lp 355, and Lc 393. At 48 h post-incubation, the live suspension monotherapy resulted in a proportional decrease in the number of viable cells according to concentrations but gemcitabine at the human plasma concentration of 50 μM outperformed it (Figure 5A). However, the combination therapy of all three strains (10^8^ CFU/well) with gemcitabine 0.1 μM reduced the number of viable cells even more than their respective monotherapeutic regimes and this reduction was similar to the group treated with the human plasma concentration of gemcitabine 50 μM (Figure 5B).

For sonicated extracts, at 48 h, only the monotherapy of Lp 355 (336 μg/mL) showed similar efficacy as gemcitabine 50 μM, while the monotherapies of Lp 12 (311 μg/mL) and Lc 393 (383 μg/mL) performed less efficacious than gemcitabine 50 μM and Lp 355 (336 μg/mL) (Figure 5C). Importantly, the combination of sonicated extracts of Lp 355 (336 μg/mL) with gemcitabine 0.1 μM inhibited CCA growth more efficiently than gemcitabine monotherapy 50 μM (Figure 5D). Lp 12 (311 μg/mL) and Lc 393 (383 μg/mL) combined with gemcitabine 0.1 μM were slightly less efficient than gemcitabine monotherapy 50 μM (Figure 5D). Interestingly, the combinations of Lp 12 (155.5 μg/mL) and Lp 355 (168 μg/mL) with gemcitabine 0.1 μM inhibited CCA growth more efficient than gemcitabine monotherapy 50 μM (Figure 5D). The combination of sonicated extracts of all three strains with gemcitabine 0.5 μM was less efficient than gemcitabine monotherapy 50 μM (Figure 5D). It is also important to mention, that combination therapy was in all strains more efficient than monotherapeutic regimes (Figure 5D).

#### 3.1.4 Live suspension or sonicated extracts of lactobacilli monotherapy and their combination with gemcitabine induced cellular senescence in CCA cell lines

**Figure 6:**
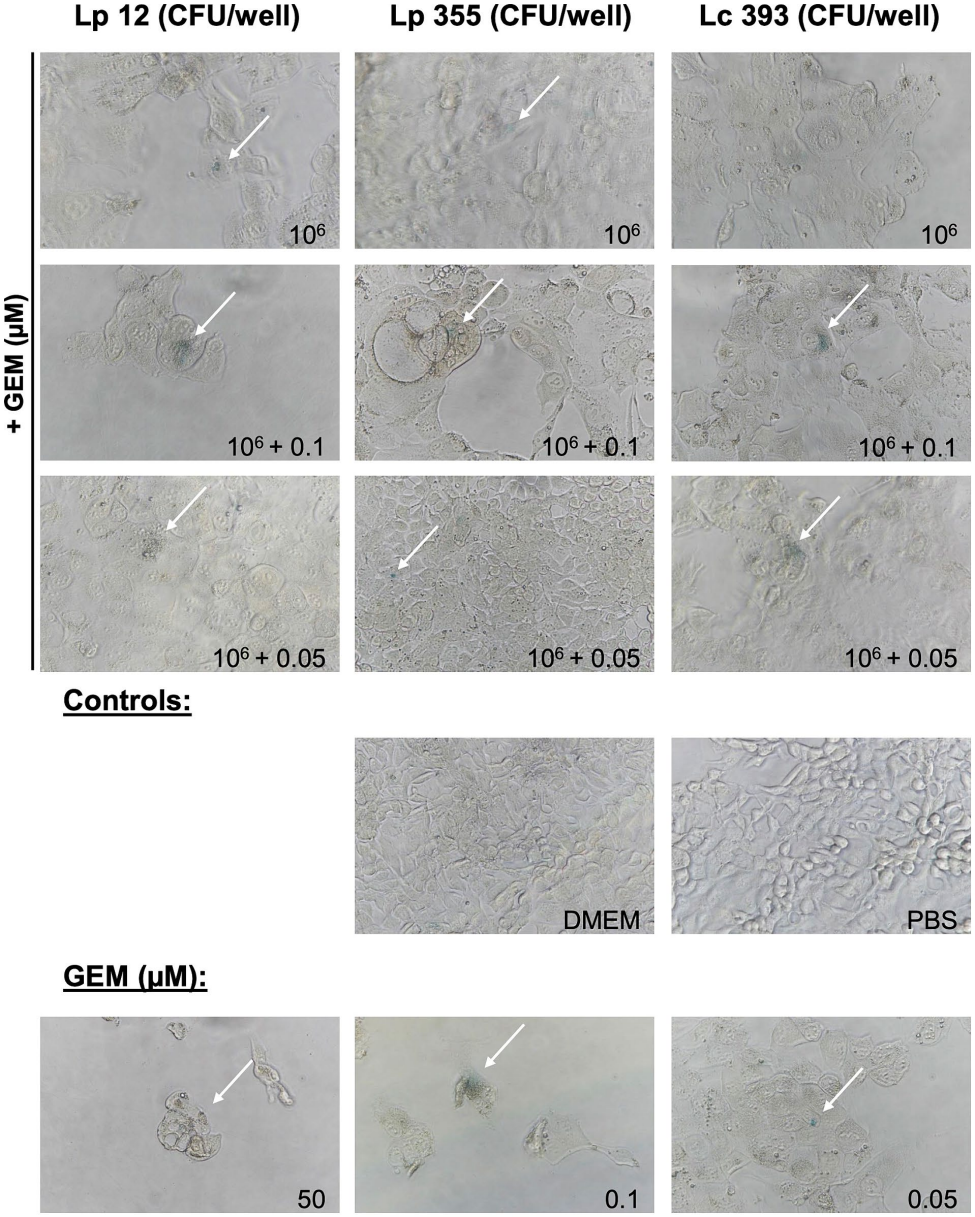
Live suspensions Lp 12, Lp 355 and Lc 393 and their combination with gemcitabine induced cellular senescence. SA-β-Gal assay was performed 48 h post-incubation. Shown are representative bright field microscopy pictures (objective 40x). Senescent (blue) cells are depicted with the white arrows.

**Figure 7:**
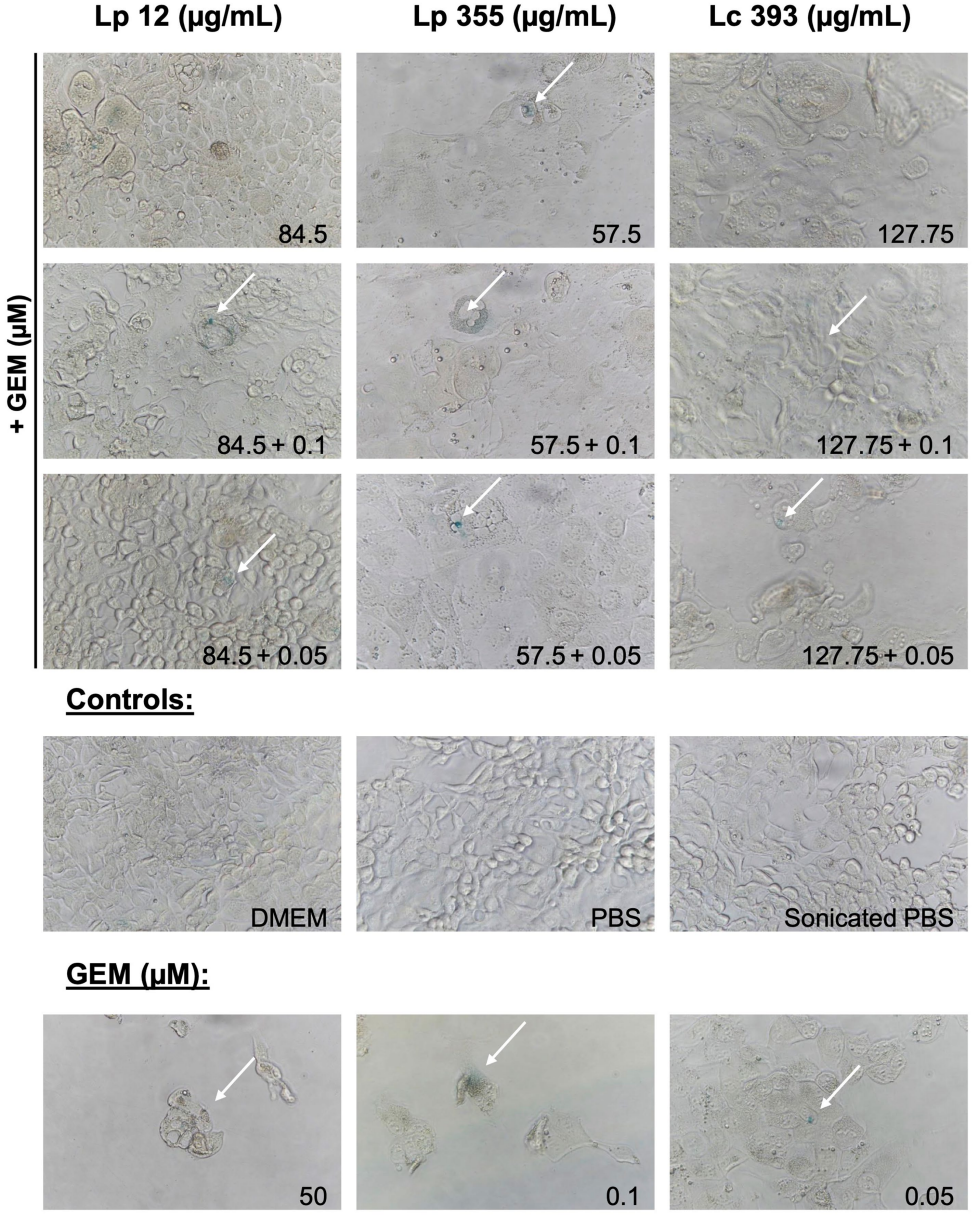
Sonicated extracts Lp 12, Lp 355 and Lc 393 and their combination with gemcitabine induced cellular senescence. SA-β-Gal assay was performed 48 h post-incubation. Shown are representative bright field microscopy pictures (objective 40x). Senescent (blue) cells are depicted with the white arrows.

We further proceeded in our experiment to confirm whether the inhibitory property of lactobacilli is associated with the induction of cellular senescence in CCA cells. We performed SA-β-Gal staining assay to detect senescent cells 48 h after treatment. The live suspensions of all three strains tested at 10^6^ CFU/well were able to induce senescence (Figure 6). Likewise, the sonicated extracts of Lp 12, Lp 355, and Lc 393 at the concentrations of 84, 57.5, and 127.75 μg/mL, respectively, induced senescence (Figure 7). The detected senescence response was comparable to the ones detected in gemcitabine of different concentrations (Figures 6, 7). Similarly, the combination therapy comprising lactobacilli with gemcitabine at 0.1 and 0.05 μM also induced senescence (Figures 6, 7).

#### 3.1.5 Lactobacilli induced early apoptosis while inhibiting CCA cell lines

**Figure 8:**
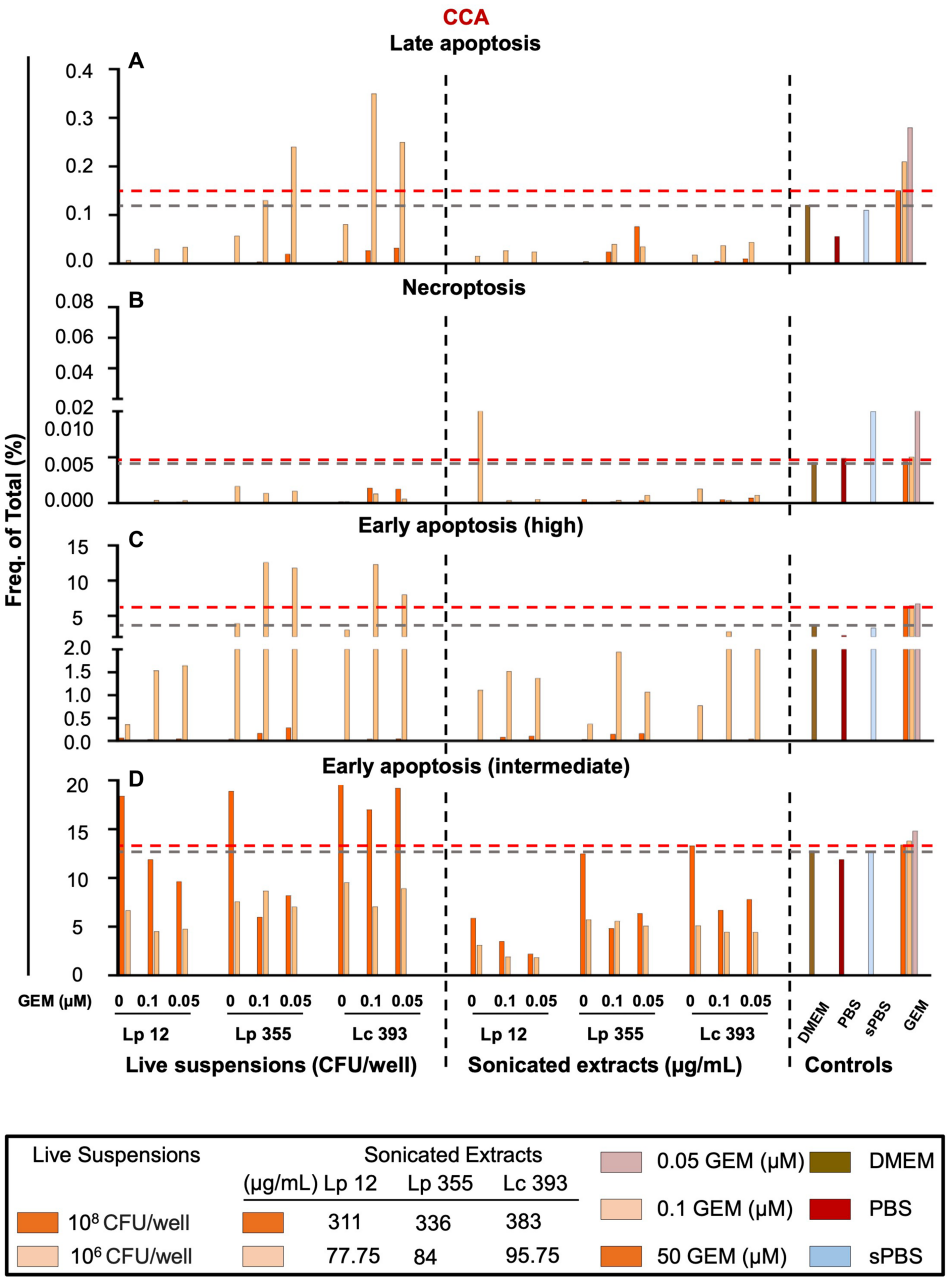
Live suspension Lp 355 induced mostly early apoptosis in CCA cells. Necroptosis, late apoptosis, early apoptosis (intermediate) and early apoptosis (high) were analyzed using the gating strategy depicted in Supplementary Figure S3. Cells were gated using forward- and side-scatter characteristics while avoiding duplicates. The results of FACS analysis showing the frequency of total cells in percent of **(A)** late apoptosis, **(B)** necroptosis, **(C)** early apoptosis (high), and **(D)** early apoptosis (intermediate) populations. GEM, gemcitabine. The red dashed line shows the data obtained in the control group treated with the standard therapy (gemcitabine 50 μM).

In our next step, we performed FACS analysis to further investigate the mechanism behind the inhibition of CCA cells. We employed a gating strategy shown in Supplementary Figure S3. Based on the gating strategy, we detected four populations: (1) early apoptosis intermediate, characterized as 7AAD^−^ Annexin V PE^intermediate^; (2) early apoptosis high, characterized as 7AAD^−^ Annexin V PE^high^; (3) late apoptosis, characterized as 7AAD^+^ Annexin V PE^+^; and (4) necroptosis, characterized as 7AAD^+^ Annexin V PE^−^ (Supplementary Figure S3). As expected, the standard therapy (gemcitabine monotherapy 50 μM) induced mostly early (intermediate and high) apoptosis in CCA (Figures 8A–D). The monotherapy of sonicated extracts Lp 12, Lp 355, and Lc 393 induced early apoptosis intermediate in a dose-dependent manner (Figure 8D). However, monotherapy of live suspension Lp 12, Lp 355, and Lc 393 induced the strongest early apoptosis intermediate even more than gemcitabine monotherapy at 50 μM (Figure 8D). Little or no cells were seen in the late apoptosis; necroptosis phase and early apoptosis-high (Figures 8A–D, respectively) in cells treated with bacterial live suspension or sonicated extracts. The combination therapy of live suspension Lp 355 and Lc 393 with 0.1 gemcitabine induced the strongest early apoptosis high (Figure 8C) and the combination therapy of live suspension Lc 393 with 0.1 μM gemcitabine induced the strongest late apoptosis (Figure 8A).

### 3.2 Anticancer activity screening in HCC cells

#### 3.2.1 Live suspension and sonicated extracts of Lp 12, Lp 355 and Lc 393 monotherapy inhibited the growth of HCC

We further tested the efficacy of lactobacilli in HCC cell line using the same experimental layout (Figure 1). In HCC settings, the live suspension at the highest concentration of 10^8^ CFU/well in all three strains, Lp 12, Lp 355, and Lc 393, proved to be more effective than the standard chemotherapy for HCC, sorafenib, at human plasma concentration (13.8 μM) (Supplementary Figures S4A–D). For sonicated extracts, Lp 12 (509, 254.5 μg/mL), Lp 355 (714, 357, 178.5 μg/mL), and Lc 393 (712, 356, and 178 μg/mL) exhibited a complete inhibition of HCC cell line growth when used as monotherapy even more than monotherapy with sorafenib at human plasma concentration (13.8 μM) (Supplementary Figures S5A–D). Further, a combination with sorafenib at concentrations 6.9 and 3.45 μM demonstrated a comparable inhibitory effect, as monotherapy, especially after 48 h of incubation (Supplementary Figures S4C,D).

Heat-killed suspensions of all strains did not demonstrate an inhibitory effect on HCC cells (Supplementary Figures S6A–D). The HCC growth was almost similar to the growth detected in negative control groups.

#### 3.2.2 A combination of live suspension or sonicated extracts of Lp 12, Lp 355, and Lc 393 with sorafenib inhibited HCC growth

We further tested cell viability using CCK-8 assay in HCC upon lactobacilli treatment. Lp 12, Lp 355 inhibited HCC cell growth in the two highest concentrations 10^7^ CFU/well and 10^8^ CFU/well, even more than caused by sorafenib at human plasma concentration (13.8 μM) (Supplementary Figure S7A). Whereas Lc 393 showed similar effect as sorafenib 13.8 μM at concentration 10^8^ CFU/well (Supplementary Figure S7A). In the case of combination therapy all three strains with either sorafenib 6.9 or 3.45 μM inhibited the growth of HCC more efficient than sorafenib 13.8 μM (Supplementary Figure S7B).

The sonicated extracts of Lp 12, Lp 355, and Lc 393 at 48 h as monotherapy or in combination performed better than live suspension and even sorafenib at human plasma concentration 13.8 μM (Supplementary Figures S7C,D).

#### 3.2.3 Live suspension of Lp 12 in monotherapy and in combination with sorafenib induced cellular senescence in HCC cell line

We aimed further to test, whether lactobacilli alone or in combination with sorafenib might result in cellular senescence induction and performed a SA-β-Gal staining in the HCC cell line after the treatments. The live suspension of Lp 12 induced senescence in HCC cells at a concentration of 10^8^, 10^7^, and 10^6^ CFU/well and also in combination with sorafenib at 6.9 μM (Supplementary Figure S8). The detected senescence response was comparable to the ones detected in sorafenib of different concentrations. Similarly, the combination therapy with sorafenib also induced senescence (Supplementary Figure S8). Senescent cells were absent in all negative controls: PBS, sonicated PBS, and DMEM.

## 4 Discussion

In this study, we investigated the anti-cancer efficacy of two strains of *L. plantarum*, Lp 12 and Lp 355 in the two prominent types of PLC, HCC, and CCA, *in vitro.* The strains were administered as live suspensions, or sonicated extracts, or heat-killed suspensions. The treatments were used either as monotherapy or as combination therapy with the standard therapeutics, sorafenib and gemcitabine and the plasma concentration of both chemotherapeutics used as control, respectively. The obtained results were compared to the classical probiotic strain *L. casei* (Lc 393).

The findings of this study revealed the significant inhibitory effect of sonicated extracts and live suspensions of Lp 12, Lp 355, and Lc 393 on HCC and CCA cell lines which provides valuable insights into the potential therapeutic application as an alternative or complementary therapy. The CVSA result of our investigation has shown that the live suspension of Lp 12, Lp 355, and Lc 393 at a concentration of 10^8^ CFU/mL is capable of inhibiting the growth of both CCA and HCC after 48 h of treatment even better than gemcitabine and sorafenib at the respective human plasma concentration. Also, the sonicated extracts of Lp 12 at 311 μg/mL, Lp 355 at 336 μg/mL, and Lc 393 at 383 μg/mL and 191.5 μg/mL were able to inhibit the growth of CCA. In the case of HCC, the sonicated extracts of all three strains were able to inhibit HCC growth although with a corresponding effect on fibroblasts.

Importantly, sonicated extracts of *L. plantarum* were more effective than the live suspension as proven in our results. The impact of the sonicated extracts of *L. plantarum* on HCC and CCA depended on concentration - higher concentrations could inhibit cell proliferation and even lead to early apoptosis; however, under low concentration, the efficacy is limited. These results suggest the optimal concentration of the *L. plantarum* sonicated extracts that is essential to inhibit cancer cell growth. The sonicated extract contains a complex mixture of different components including cell walls, peptidoglycan, and several other proteins (Han et al., 2013). Various researches on the potential effect of different components of the sonicated extracts on different diseases were described, however, there is no extensive research conducted on the effect on cancer cells. The sonicated extracts of *L. reuteri* have been reported to enhance the wound healing process by regulating PI3K/AKT/ß-catenin/TGFß1 pathways (Han et al., 2019). Similarly, nisin which is a protein present in the cytoplasmic extract of sonicated *Lactococcus lactis* subsp. *lactis* and its cell wall have antiproliferative and antitumor effects by decreasing cyclin D1 in SW480 colon cancer cell lines (Hosseini et al., 2020). The inhibitory potency displayed by our sonicated extracts of lactobacilli on HCC and CCA is not farfetched as it correlates and agrees with other previous research studies highlighted above. For the future, we plan to perform a liquid chromatography with tandem mass spectrometry to fractionate the composition of the sonicated extracts and test effect of individual components on CCA and HCC cell line to identify the most potent metabolites.

It was highly unexpected that heat-killed suspensions demonstrated a total lack of capacity to inhibit the growth of HCC and CCA cell lines. It could be that the heat-killed suspensions may possess some growth factors which might have allowed or even enhance the growth of cancer cells. The heat-killed suspensions component may include cell wall components, lipoteichoic acids, peptidoglycan, surface proteins, and polysaccharides (Aiba et al., 2017). In contrast to our results, the whole peptidoglycan extracted from *L. paracasei* was reported to have cytotoxic activity against colon cancer HT-29 cells by upregulating proapoptotic genes and downregulating antiapoptotic genes (Wang et al., 2018). In another study where the anti-tumor effect of heat-killed *L. reuteri* and *L. casei* was tested on human colorectal carcinoma, it was proven that the extracts have a moderate cytotoxic and apoptotic effect *in vitro* as against when it was orally administered to a xenograft model bearing RKO cells (Kim et al., 2022). Also, the bacteria cells were heat-killed at 100°C for 30 min as against our regimen of 95°C for 1 h. This could have impacted the disparity in the results obtained. Our data *in vitro* is further supported by the *in vivo* data of Si et al., who showed that an oral administration of heat-killed *L. rhamnosus* monotherapy failed to reduce the tumor volume in MC38 tumor-bearing mice and even failed to improve the efficacy of anti-PD-1 antibody treatment when combined (Si et al., 2022). All these suggest that the anticancer property of heat-killed suspensions vary among different species of *Lactobacillus*, the route of administration, the conditions of heat inactivation, and differences in cell lines.

Furthermore, in our results, the CCK-8 assay clearly showed that the combination therapy of live suspension and sonicated Lp 12, Lp 355, and Lc 393 with 0.1 μM gemcitabine displayed a clear inhibitory effect in CCA more than their individual and gemcitabine monotherapy. In the same vein, mostly the sonicated extracts of Lp 12, Lp 355, and Lc 393 were able to have an inhibitory effect more than sorafenib monotherapy. This result proves our hypothesis that combination therapy will be more beneficial and will reduce the side effects of standard chemotherapeutics because it will allow usage at a reduced concentration. The sonicated extracts or live suspension of Lp 12, Lp 355, and Lc 393 can be used as a complementary therapy to reduce the side effects of sorafenib. A combination therapy that will ensure optimal efficacy with a reduced dosage of standard chemotherapy will be a promising approach in cancer treatment. Sorafenib, which is the standard chemotherapeutic for HCC has a lot of adverse effects which include hand-foot skin reaction, rash, hypertension, diarrhea, fatigue, anorexia, thrombocytopenia, alopecia, and bilirubin elevation (Cheng et al., 2009; Llovet et al., 2008). On the other hand, gemcitabine, the standard chemotherapeutic for CCA is also associated with some adverse reactions such as myelosuppression, pulmonary toxicity, and capillary leak syndrome (Mertz et al., 2019; Turco et al., 2015). In previous studies, there are reports that the gut microbiota possesses the ability to modulate the efficacy and toxic profile of cancer chemotherapy: the gut microbiota after treatment with *L. rhamnosus* GG supplement was able to reduce chemotherapy of colorectal cancer-associated diarrhea and abdominal discomfort with no lactobacillus-related toxicity (Alexander et al., 2017). Also, another study by Rodriguez-Arrastia et al. highlighted the significance of probiotic supplements mainly of *Lactobacillus* species in addressing the related side effects caused by varying treatments in oncology patients (Rodriguez-Arrastia et al., 2021). Also, Rodriguez-Arrastia et al. studies substantiate the hypothesis and suggest that combination therapy will provide more benefit than monotherapy because it combines both the curative effect of standard chemotherapeutics and lactobacilli (Rodriguez-Arrastia et al., 2021).

Based on our results, we further were able to ascertain that the live suspensions and sonicated extracts of *L. plantarum* were able to induce cellular senescence. Cellular senescence is a stable exit from the cell cycle caused by different intrinsic and extrinsic factors (Campisi and d'Adda di Fagagna, 2007), but in our research it was caused by the therapeutic effect of *L. plantarum*. Modulation of cellular senescence by probiotics especially the bacteria of the genus *Lactobacillus* has been studied extensively in the context of aging and longevity owing to their anti-inflammatory, anti-oxidant, and anti-immunosenescence properties (Sharma and Padwad, 2020). However, there is no scientific literature that has provided evidence of probiotics and their metabolites inducing cellular senescence in cancer cells. Our study shows it for the first time. Senescent cells are metabolically viable and may persist indefinitely, and are typically arrested at the G1 or G2/M phases of the cell cycle (Campisi and d'Adda di Fagagna, 2007). Interestingly, all concentrations of live suspension and sonicated extracts of Lp 12 were able to induce senescence in CCA cells and the live suspension of Lp 12 in HCC cells. However, cellular senescence induction was more apparent in the live suspension monotherapy of Lp 12 at 10^7^ CFU/well in both CCA and HCC cells, sonicated extract monotherapy of Lp 12 at 77.75 μg/mL in CCA cells, as well as the combination therapy of live suspension of Lp 12 at 10^6^ CFU/mL and 0.05 μM gemcitabine in CCA cells. The observation that lower concentrations of Lp 12 induce senescence suggests the potential benefit of therapy-induced senescence. Studies have suggested that senescent cells can facilitate the expression of cytokines and factors that may inhibit the growth of surrounding tumor cells (Di et al., 2008; Kang et al., 2011; Xue et al., 2007). Data from a recent study also demonstrated that senescent cells owing to their long-term persistence *in vivo* are strongly immunogenic, activate interferon signaling, antigen-presenting cells, CD8 T cells, and MHC class I molecule through the development of a proinflammatory secretome known as senescence-associated secretory phenotype (Eggert et al., 2016; Marin et al., 2023; Yevsa et al., 2012).

*Lactobacillus* species significantly modulate and regulate the immune system (Parada Venegas et al., 2019; Wiese-Szadkowska et al., 2019). They can enhance the proliferation of T lymphocytes and strengthen the killing ability of natural killer cells (Meng et al., 2018), enhance the phagocytic abilities of macrophages (Jung et al., 2015), and the maturation of B cells (Garcia-Castillo et al., 2019). The modulation of the immune system is essential in preventing excessive inflammation and fighting infection (Iddir et al., 2020). Furthermore, lactobacilli also help to maintain the integrity of the intestinal epithelial barrier by protecting against injuries induced by pathogenic bacteria by increasing transepithelial electrical resistance, downregulation of permeability of IL-8, and regulation of extracellular signal-regulated kinase and c-jun N-terminal kinase (Yu et al., 2015). Regarding the above-discussed health benefit of lactobacilli, it is indispensable to study its anticancer potential in PLC.

The anticancer effect of lactobacilli has been investigated in the past. *L. plantarum* has been reported to reduce oxidative stress and prevent the development and progression of cancer (Kullisaar et al., 2002; Wu et al., 2021), but a comprehensive understanding of their efficacy in PLC is needed. Most importantly, our study further unraveled the mechanism of action of lactobacilli in inhibiting the growth of PLC cells. We could prove that induction of early apoptosis was the main mechanism behind the inhibitory capacity of live suspension and sonicated extracts of lactobacilli in CCA cells. In line with the result of our study, the cytotoxicity effect of *L. plantarum* treatment on breast, colorectal, liver, and leukemia cancer cell lines was mediated by the induction of apoptosis (Chuah et al., 2019). Recently, it has been demonstrated that the live suspension of *L. paracasei* X12 had a tumor suppressive effect on colorectal cancer by preventing weight loss and decreasing tumor volume; downregulated proliferation inducer and anti-apoptotic like Bcl-3, Jak-1, and Akt-1 genes and upregulated pro-apoptotic genes like Bax, Cas-3 in a DMH-induced rat model (Jam et al., 2020). Another study reported that the live suspension of *L. casei* co-incubated with colon cancer cells resulted in decreased viability and induction of apoptosis (Tiptiri-Kourpeti et al., 2016). This was further confirmed *in vivo* after daily oral administration for 13 days (Tiptiri-Kourpeti et al., 2016). In line with our data, studies have demonstrated that *L. plantarum* induces apoptosis and prevents metastasis and invasion of cancer cells (Belury, 2002; Choi et al., 2006). Furthermore, there is an established correlation between the quality of the microbiome and tumor progression (Dzutsev et al., 2017). Dysbiosis or an imbalance in the composition of the gut microbiota has a direct negative implication on liver health by increasing metabolic disorders and the abundance of pathogenic bacteria causing inflammation and subsequently leading to chronic liver disease and progression to PLC, including HCC and CCA (Chen et al., 2020). *L. plantarum* has been shown to improve the gut microbiome and to inhibit the proliferation of potentially pathogenic bacteria (Hong et al., 2021). By restoring the healthy gut microbiota composition, *L. plantarum* can potentially mitigate the risk of liver cancer progression from dysbiosis (Yu and Schwabe, 2017). It has also been demonstrated that the soluble polysaccharides isolated from *L. acidophilus* 606 resulted in the death of HT-29 cancer cells attributed to the induction of apoptosis and antioxidative activity (Choi et al., 2006). Moreover, *L. acidophilus* has been shown to suppress metabolic dysfunction-associated steatotic malignant liver disease through producing of valeric acid (Lau et al., 2024). Similarly, mitochondrial apoptotic pathways were activated, leading to the death of colorectal C26 cancer cells when treated with *L. plantarum* suspension (Sharifi et al., 2022). The cell-free extract of *L. plantarum* tested on high and low metastatic human malignant melanoma cells on a mice model induced intrinsic apoptosis by regulating Bax/Bcl-2 ratio (Park et al., 2020). Also, Sun et al. proved that the crude exopolysaccharide of *L. plantarum* has an inhibitory effect on human colon cancer lines by increasing the pro-apoptotic protein level (Sun et al., 2021). Finally, lactobacilli were also demonstrated to cause a robust antitumor effect by reducing IL-17, an inflammatory cytokine, and other angiogenesis factors resulting in inhibition of HCC growth in mice by 40% (Li et al., 2016). Also, the positive role of lactobacilli consumption in inhibiting colorectal cancer and HCC induced in mice through the stimulation and expression of miRNAs was described (Heydari et al., 2019).

In summary, the live suspension and sonicated extracts of Lp 12 and Lp 355 demonstrated strong inhibitory properties in both CCA and HCC in a dose-dependent manner and were either comparable to, or showed even more efficacy than a classical probiotic strain Lc 393, which served as a control in this study. At the same time Lp 12 and Lp 355 showed less toxic effects on fibroblast cells, than Lc 393. Surprisingly, the heat-killed suspensions of Lp 12, Lp 355, and Lc 393 displayed no inhibitory effect in HCC and CCA. Furthermore, the combination of live suspensions or sonicated extracts of Lp 12, Lp 355, and Lc 393 with standard chemotherapeutics (sorafenib for HCC and gemcitabine for CCA) demonstrated a synergistic inhibitory effect comparing to the individual monotherapy and the human plasma concentration of sorafenib and gemcitabine. The combination therapy allows for a reduced concentration of standard chemotherapeutics, consequently savaging the adverse effect. We have also shown that Lp 12 can induce cellular senescence in both cancer cell lines which in turn could translate to the activation of immune cells. The mechanism of action of the lactobacilli was shown to be associated with the induction of early apoptosis in CCA. The most promising therapeutic candidates for CCA are the live suspensions of Lp 12 and Lp 355 at a concentration of 10^8^ CFU/mL, combination therapy of live suspension of Lp 12 and gemcitabine at 0.1 μM, and also, the sonicated extracts of Lp 12 and Lp 355 at 311 μg/mL and 336 μg/mL, respectively. The most promising therapies for HCC are the sonicated extracts of Lp 355 and Lc 393 at 178 μg/mL and in combination with sorafenib at 6.9 μM. More intensive research is needed to advance this therapy development studies and further investigate the safety, efficacy, and immune responses *in vivo* applying monotherapeutic or combination therapy regimes with *L. plantarum*in HCC and CCA using preclinical models.

## 5 Conclusion

We found that both tested *Lactobacillus* strains – *L. plantarum* ONU 12 (Lp 12) and *L. plantarum* ONU 355 (Lp 355) showed potent anti-cancerous capacity toward CCA and HCC cell lines *in vitro*. By combining these strains with traditional chemotherapy drugs, we were able to significantly reduce the concentration of gemcitabine and sorafenib. Our results suggest that the mechanism behind this successful inhibition involves the induction of early apoptosis and cellular senescence. However, *in vivo* studies are necessary to evaluate the efficacy of *L. plantarum* strains and their mechanisms of action in autochthonous HCC and CCA models. Combination therapies that demonstrate synergistic effects, thereby reducing the doses of toxic chemotherapy drugs, hold great promise for patients with HCC and CCA.

## Data Availability

The original contributions presented in the study are included in the article/Supplementary material, further inquiries can be directed to the corresponding author.
