# Supplementary material for: Lactiplantibacillus plantarum inhibited the growth of primary liver cancer by inducing early apoptosis and senescence, in vitro
Source: Front Microbiol. 2024 Nov 11;15:1451170. doi: 10.3389/fmicb.2024.1451170 (PMC11590124; doi:10.3389/fmicb.2024.1451170)
Supplement: Supplementary file 1 [file Data_Sheet_1.pdf]

Supplementary Material

Lactiplantibacillus plantarum inhibited the growth of primary liver cancer by inducing early apoptosis and senescence, *in vitro*.

Oladimeji Paul Duduyemi[1§], Kateryna Potapenko[1, 2§], Nataliia Limanska[2], Sofiya Kotsyuda[1], Nataliia Petriv[1], Huizhen Suo[1], Tetyana Gudzenko[2], Volodymyr Ivanytsia[2], Tetyana Yevsa[1*]

[1]Department of Gastroenterology, Hepatology, Infectious Diseases, and Endocrinology, Hannover Medical School, Hannover, Germany

[2]Department of Microbiology, Virology, and Biotechnology, Odesa I. I. Mechnykov National University, Odesa, Ukraine

[§]These authors contributed equally.

[*]**Correspondence:**
Tetyana Yevsa, PhD
Department of Gastroenterology, Hepatology, Infectious Diseases and Endocrinology
Hannover Medical School
Carl-Neuberg Str. 1
30625 Hannover, Germany
Tel.: +49 (0) 511 532 83164
Fax: +49 (0) 511 532 5692
E-mail address: Yevsa.Tetyana@mh-hannover.de

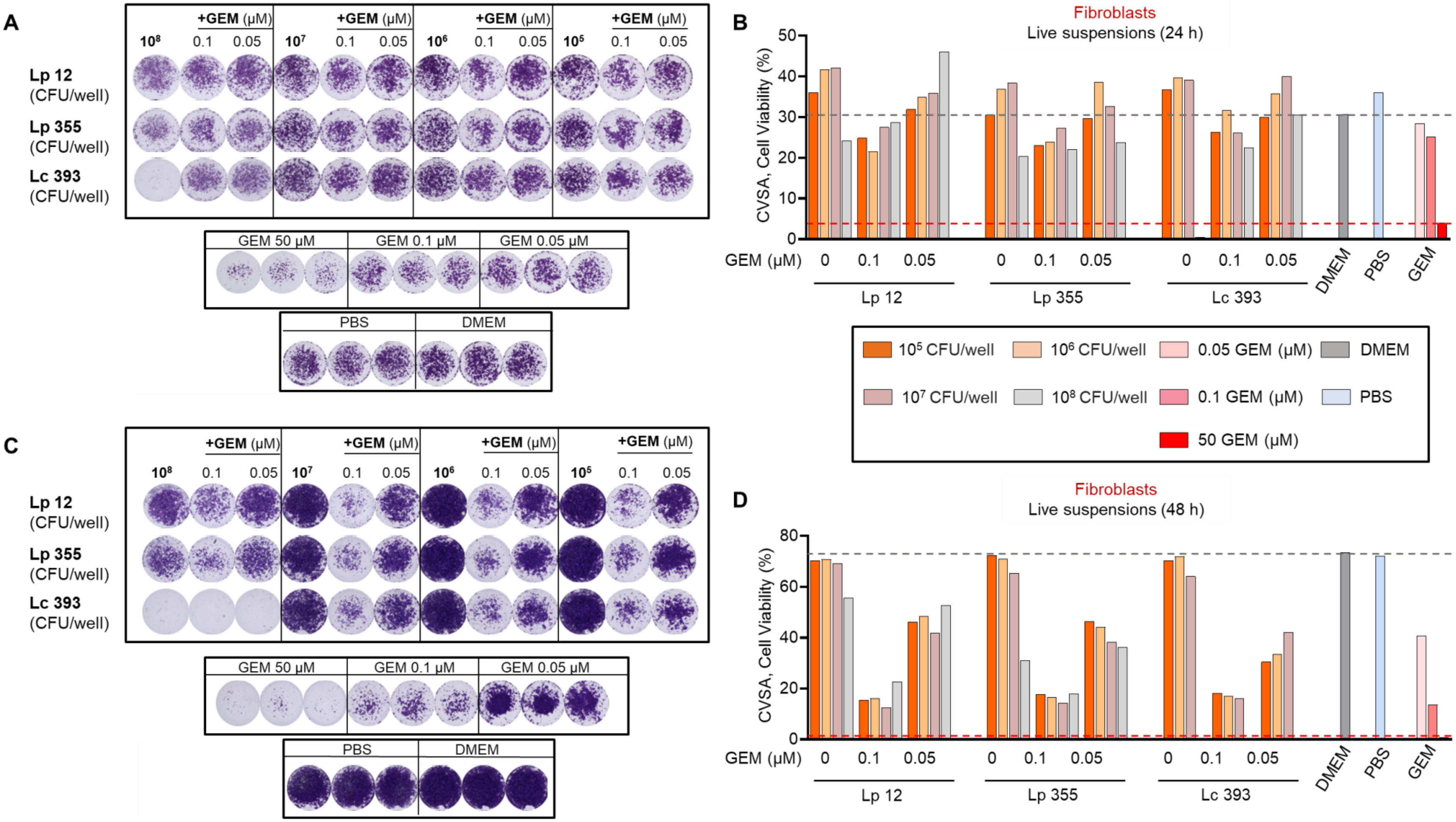

Supplementary Figure 1. The live suspension of Lc 393 at the concentration of 10^8 CFU/well inhibited the growth of fibroblasts. CVSA analysis was performed 24 h and 48 h post-incubation. Data are depicted as microphotographs shown in **(A)** 24 h and **(C)** 48 h post-incubation

as well as graphical representation **(B)** 24 h and **(D)** 48 h post-incubation. GEM = gemcitabine. The red dashed line shows the data obtained in the control group treated with the standard therapy (gemcitabine 50 μM).

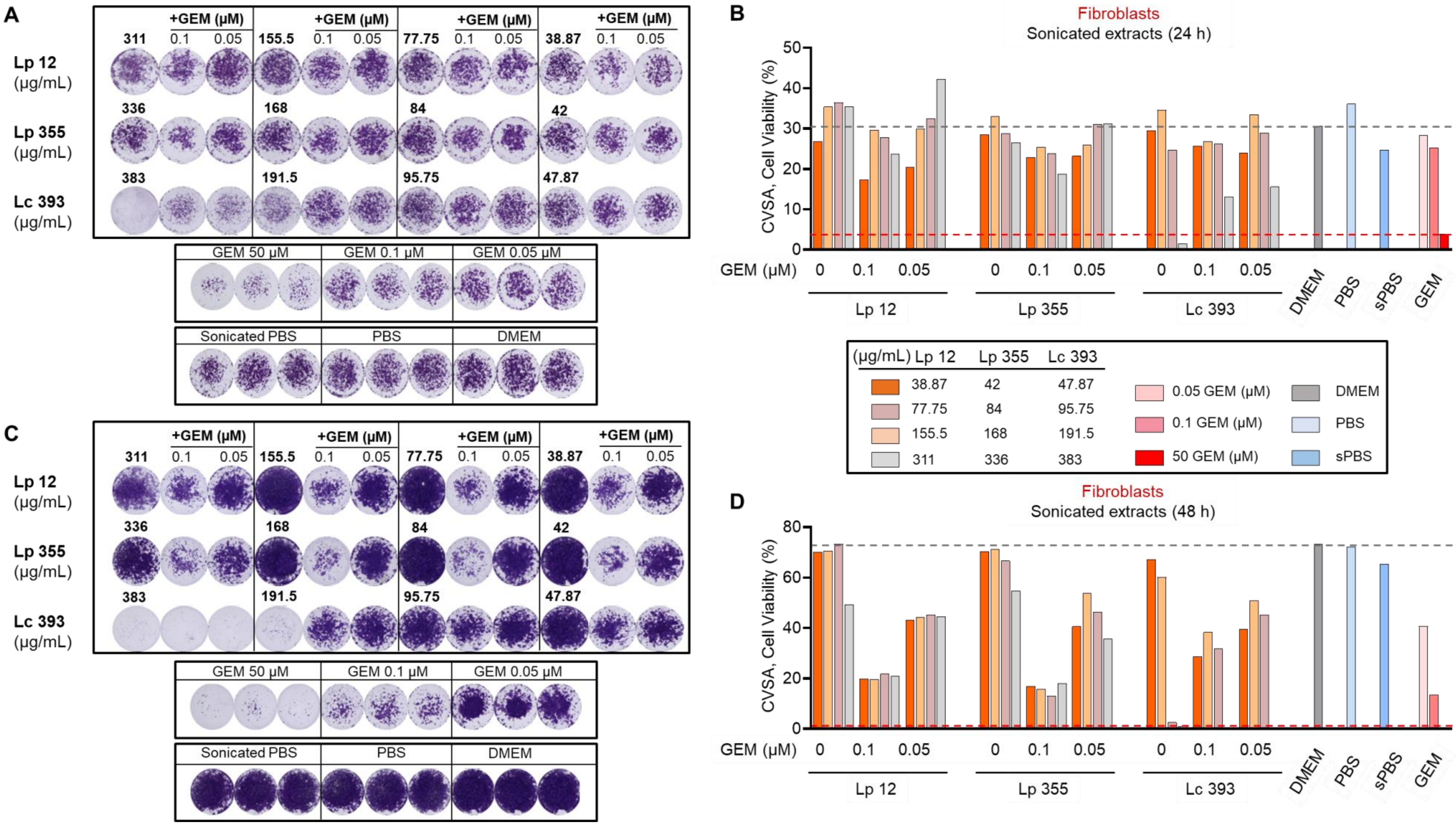

Supplementary Figure 2. The sonicated extract of Lc 393 at the concentrations 383 and 191.5 μg/mL inhibited the growth of fibroblasts. CVSA analysis was performed 24 h and 48 h post-incubation. Data are depicted as microphotographs shown in **(A)** 24 h and **(C)** 48 h post-incubation as well as graphical representation **(B)** 24 h and **(D)** 48 h post-incubation. GEM = gemcitabine, sPBS = sonicated PBS. The red dashed line shows the data obtained in the control group treated with the standard therapy (gemcitabine 50 μM).

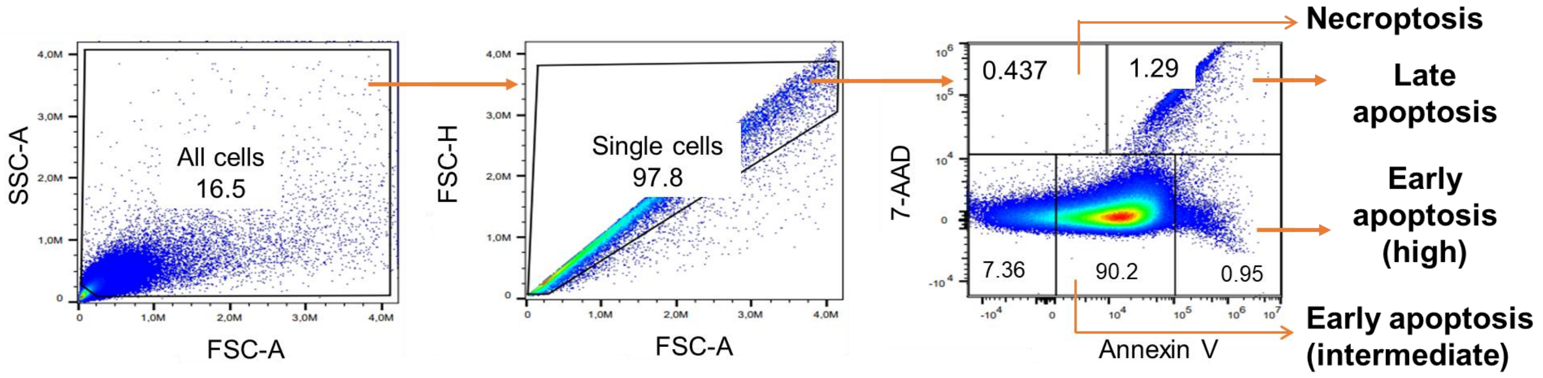

Supplementary Figure 3. Gating strategy used in FACS analysis.

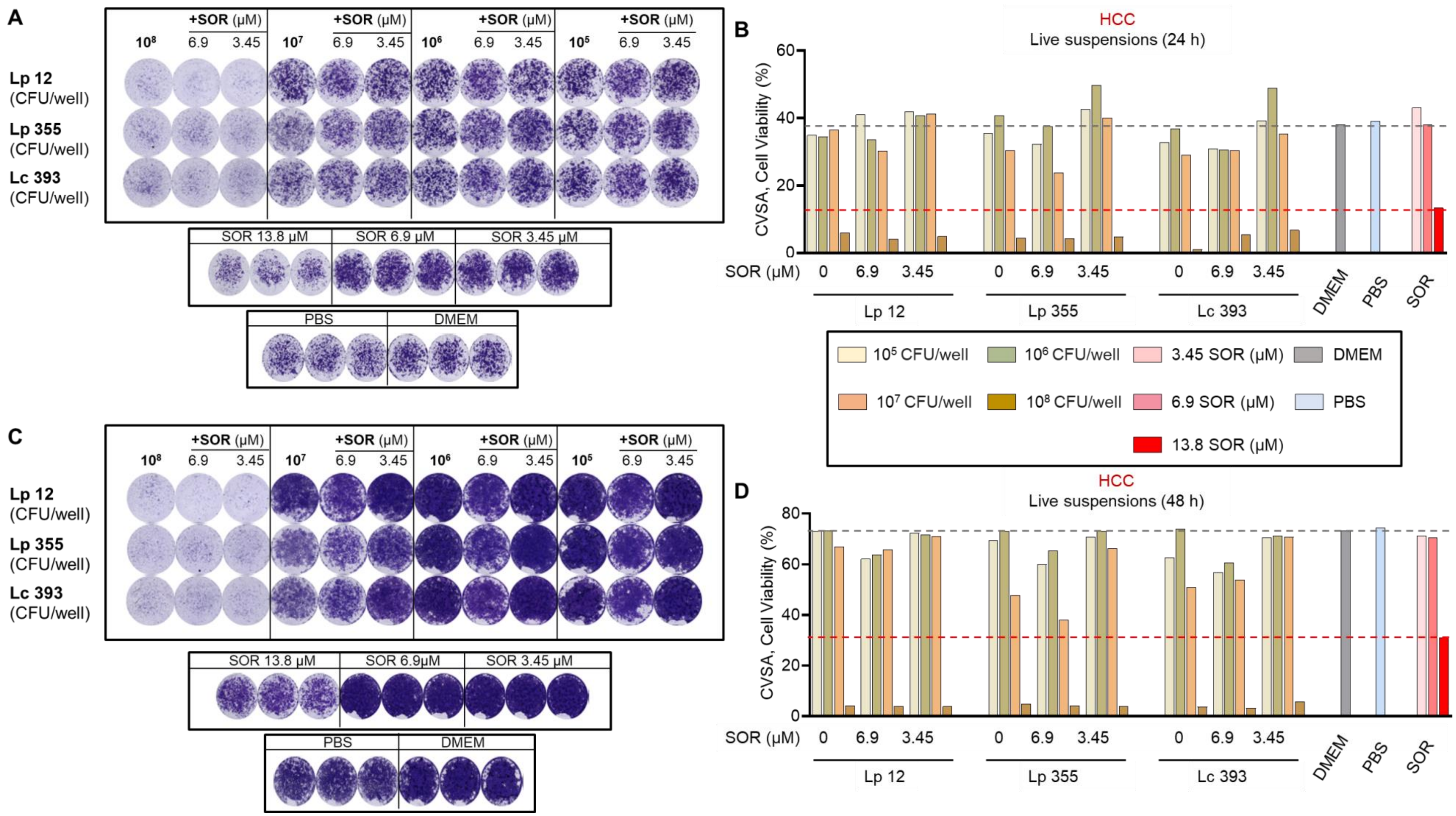

Supplementary Figure 4. The live suspension of Lp 12, Lp 355, and Lc 393 at the 10^8 CFU/well concentration efficiently inhibited the growth of HCC cells. CVSA analysis was performed 24 h and 48 h post-incubation in HCC cells after the treatment with lactobacilli live suspension either alone at different concentrations or in combination with sorafenib at different concentrations (3.45 and 6.9 μM). Data are depicted as microphotographs shown in **(A)** 24 h and **(C)** 48 h post-incubation as well as graphical representation **(B)** 24 h and **(D)** 48 h post-incubation. SOR = sorafenib. The red dashed line shows the data obtained in the control group treated with the standard therapy (sorafenib 13.8 μM).

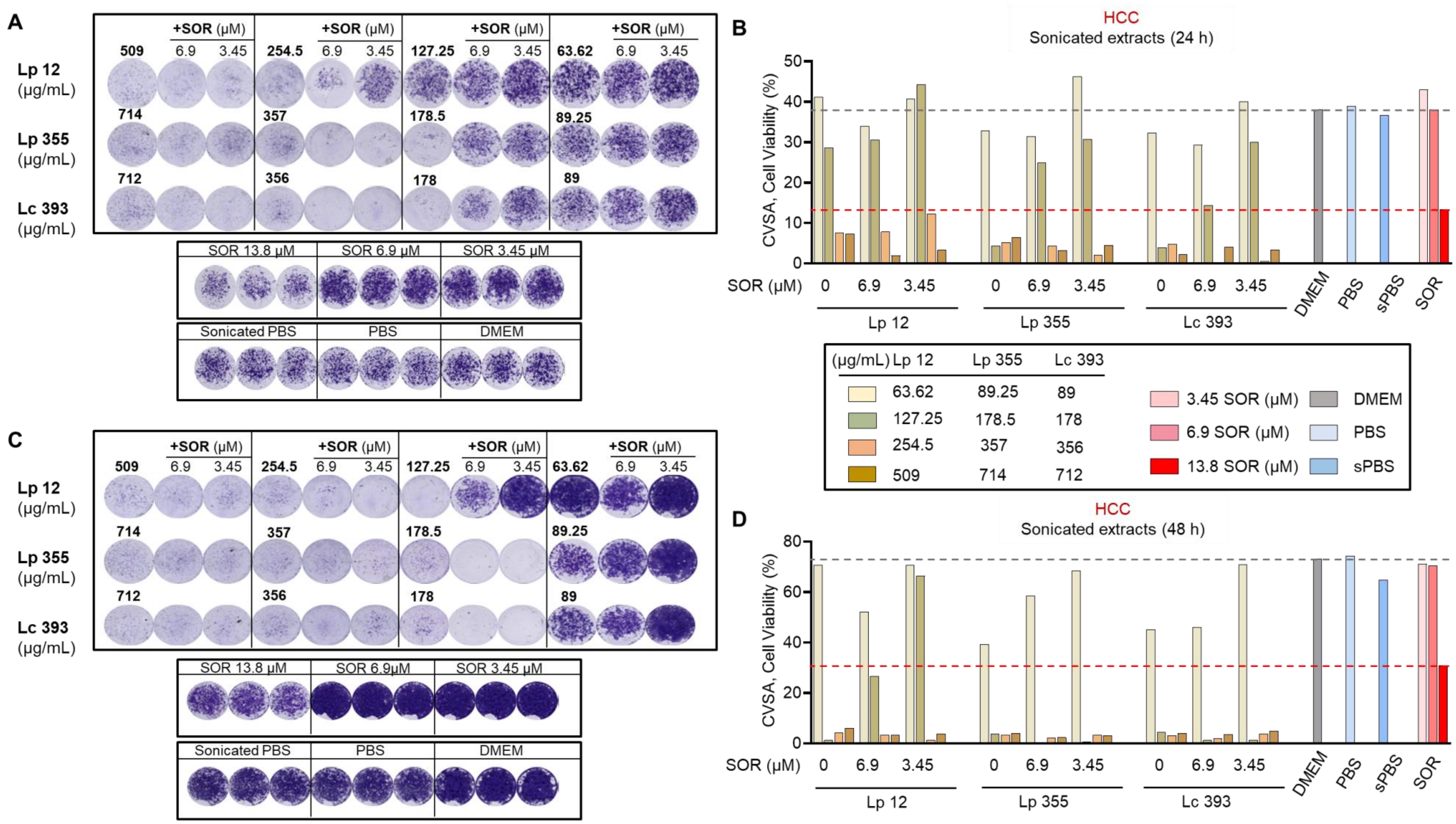

Supplementary Figure 5. The sonicated extracts of Lp 12, Lp 355 and Lc 393 and their combination with sorafenib inhibited the growth of HCC cells. CVSA analysis was performed 24 h and 48 h post-incubation in HCC cells after the treatment with lactobacilli sonicated extracts either alone at different concentrations or in combination with sorafenib at different concentrations (3.45 and 6.9 µM). Data are depicted as microphotographs shown in **(A)** 24 h and **(C)** 48 h post-incubation as well as graphical representation **(B)** 24 h and **(D)** 48 h post-incubation. SOR = sorafenib, sPBS = sonicated PBS. The red dashed line shows the data obtained in the control group treated with standard therapy (sorafenib 13.8 µM).

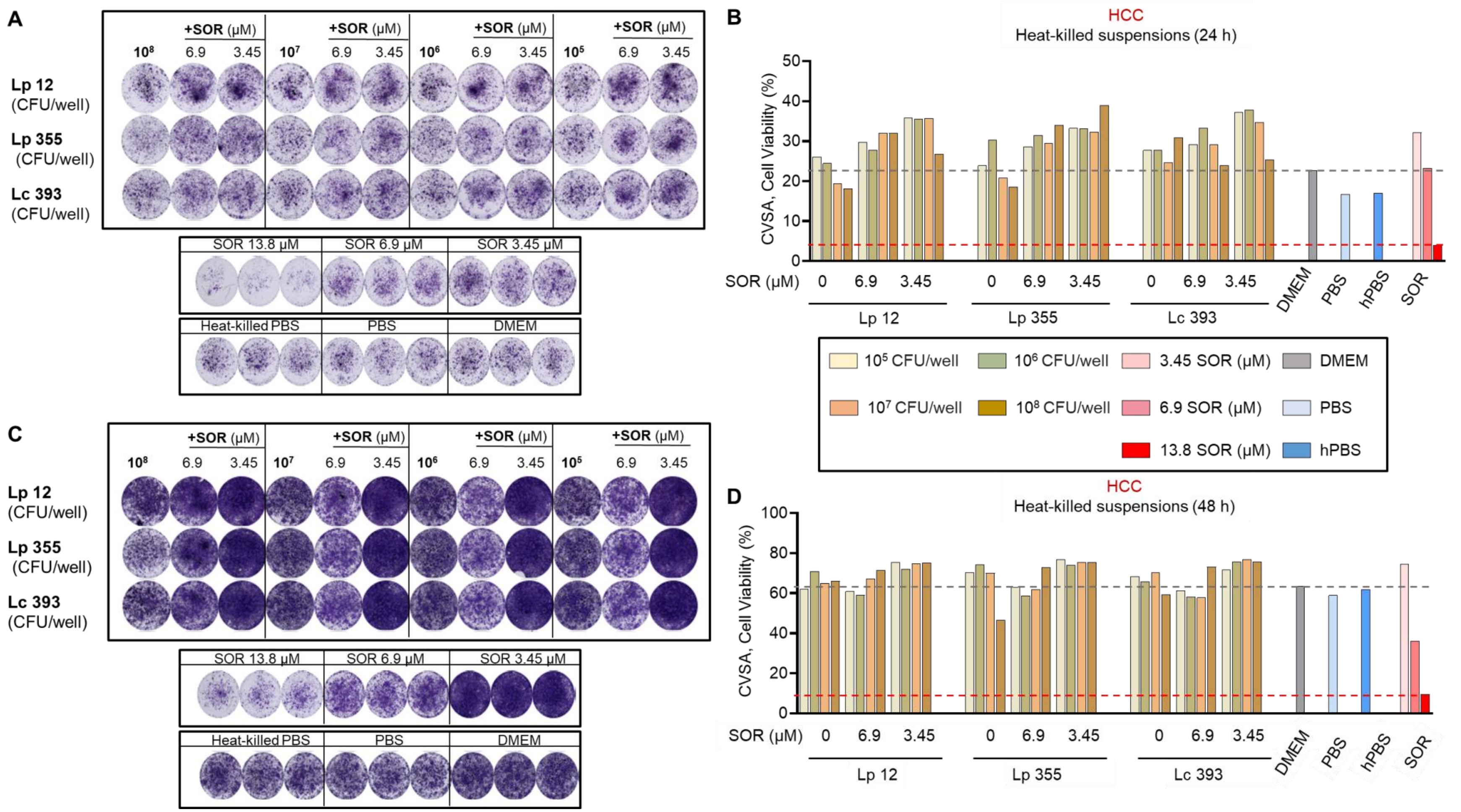

Supplementary Figure 6. The heat-killed suspensions of Lp 12, Lp 355 and Lc 393 did not show any inhibitory activity in HCC cell line. CVSA analysis was performed 24 h and 48 h post-incubation in HCC cells after the treatment with heat-killed lactobacilli either alone at different concentrations or in combination with sorafenib at different concentrations (3.45 and 6.9 μM). Data are depicted as microphotographs shown in **(A)** 24 h and **(C)** 48 h post-incubation as well as graphical representation **(B)** 24 h and **(D)** 48 h post-incubation. SOR = sorafenib. hPBS = heated PBS. The red dashed line shows the data obtained in the control group treated with the standard therapy (sorafenib 13.8 μM).

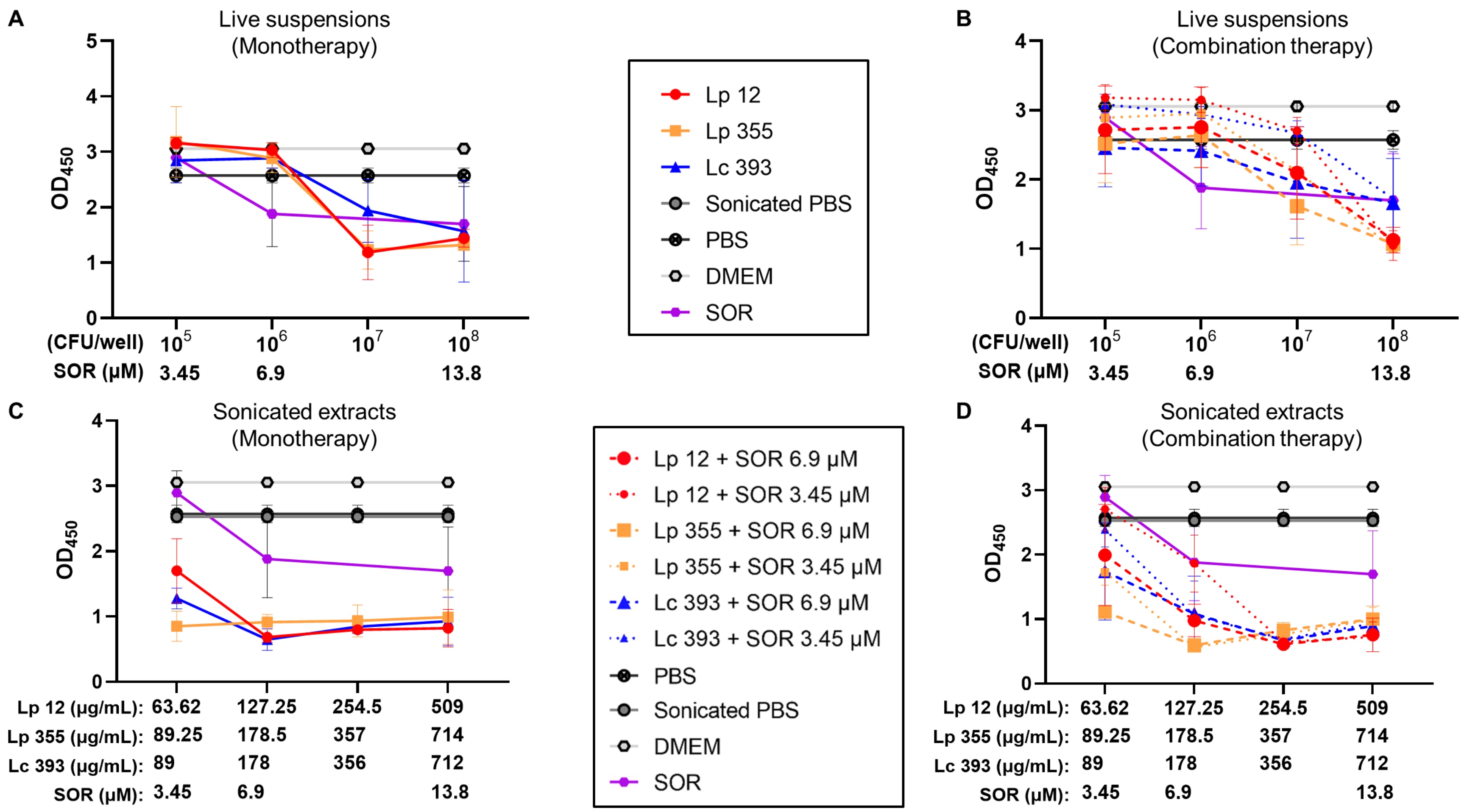

Supplementary Figure 7. Sonicated extracts of Lp 12, Lp 355, and Lc 393 and their combination with sorafenib demonstrated inhibitory effects on HCC cell line in CCK-8 analysis. HCC cells were treated with different formulations of lactobacilli and their combination with sorafenib. CCK-8 analysis was performed 48 h post-incubation using OD_{450} on HCC cells treated with: **(A)** live suspension lactobacilli monotherapy; **(B)** live suspension in combination with sorafenib; **(C)** sonicated extract lactobacilli monotherapy; **(D)** sonicated extract lactobacilli in combination with sorafenib.

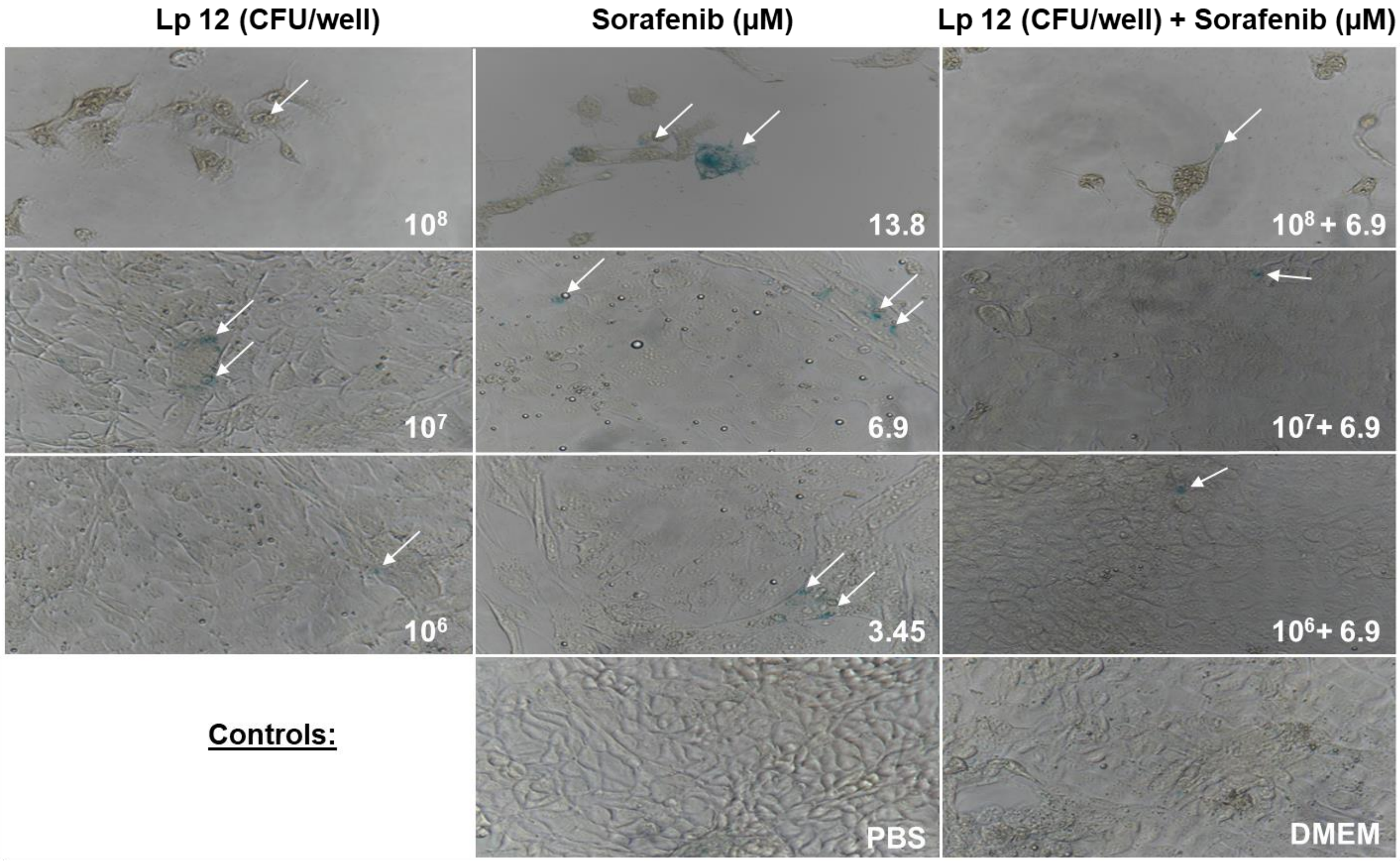

Supplementary Figure 8. Live suspension of Lp 12 and it‘s combination with sorafenib induced cellular senescence in HCC cell line. SA-β-Gal assay was performed 48 h post-incubation. Shown are representative bright field microscopy pictures (objective 40x). Senescent (blue) cells are depicted with the white arrows.
